# Developing an Evidence- and Theory-Informed Mother-Daughter mHealth Intervention Prototype Targeting Physical Activity in Preteen Girls of Low Socioeconomic Position: Multiphase Co-Design Study

**DOI:** 10.2196/62795

**Published:** 2025-01-06

**Authors:** Carol Brennan, Grainne ODonoghue, Alison Keogh, Ryan E Rhodes, James Matthews

**Affiliations:** 1 School of Public Health, Physiotherapy and Sports Science University College Dublin Dublin 4 Ireland; 2 School of Medicine Trinity College Dublin Dublin Ireland; 3 Behavioural Medicine Laboratory University of Victoria Victoria, BC Canada

**Keywords:** physical activity, preteen girls, socioeconomic position, maternal support, mHealth, intervention, co-design, pediatric, daughter, design, development, behavior change technique, Behaviour Change Wheel, sedentary, inactivity

## Abstract

**Background:**

Preteen girls of lower socioeconomic position are at increased risk of physical inactivity. Parental support, particularly from mothers, is positively correlated with girls’ physical activity levels. Consequently, family-based interventions are recognized as a promising approach to improve young people’s physical activity. However, the effects of these interventions on girls’ physical activity are often inconsistent, with calls for more rigorous, theory-informed, and co-designed family-based interventions to promote physical activity in this cohort.

**Objective:**

This study aimed to use co-design methods to develop an evidence- and theory-informed mother-daughter mobile health intervention prototype targeting physical activity in preteen girls.

**Methods:**

The intervention prototype was developed in accordance with the United Kingdom Medical Research Council framework, the Behaviour Change Wheel, the Theoretical Domains Framework, and the Behaviour Change Techniques Ontology. The Behaviour Change Intervention Ontology was also used to annotate the intervention characteristics. The co-design process incorporated three phases: (1) behavioral analysis, (2) the selection of intervention components, and (3) refinement of the intervention prototype. Throughout these phases, workshops were conducted with preteen girls (n=10), mothers of preteen girls (n=9), and primary school teachers (n=6), with additional input from an academic advisory panel.

**Results:**

This 3-phase co-design process resulted in the development of a theory-informed intervention that targeted two behaviors: (1) mothers’ engagement in a range of supportive behaviors for their daughters’ physical activity and (2) daughters’ physical activity behavior. Formative research identified 11 theoretical domains to be targeted as part of the intervention (eg, knowledge, skills, and beliefs about capabilities). These were to be targeted by 6 intervention functions (eg, education, persuasion, and modeling) and 27 behavior change techniques (eg, goal setting and self-monitoring). The co-design process resulted in a mobile app being chosen as the mode of delivery for the intervention.

**Conclusions:**

This paper offers a comprehensive description and analysis of using co-design methods to develop a mother-daughter mobile health intervention prototype that is ready for feasibility and acceptability testing. The Behaviour Change Wheel, Theoretical Domains Framework, and Behaviour Change Techniques Ontology provided a systematic and transparent theoretical foundation for developing the prototype by enabling the identification of potential pathways for behavior change. Annotating the Behaviour Change Intervention Ontology entities represents the intervention characteristics in a detailed and structured way that supports improved communication, replication, and implementation of interventions.

## Introduction

### Background

Globally, 81% of adolescents are not meeting the recommended physical activity (PA) guidelines [1], with PA levels regressing annually throughout adolescence [2,3]. This rate of decline is more pronounced in girls than boys [1,4] and is most apparent during the transition period from primary to secondary school [5,6]. Studies also indicate that children of lower socioeconomic position (SEP) are less likely to be physically active than those of higher SEP [7-9]. Indeed, this is noteworthy in girls of low SEP, as evidence indicates that this cohort experiences a steeper decline in PA than their more advantaged peers at the transition to adolescence [4,9], putting them at a greater risk of obesity, type 2 diabetes, and cardiovascular disease [8,10]. Most interventions targeting children’s daily PA levels have taken place during school hours [11]; however, children are reported to be less active during time spent outside of school, such as at weekends or holidays [12]. Thus, there is a need to also promote PA outside of the school context [13].

Families are a central foundation of support and guidance for children and adolescents in shaping healthy PA behaviors particularly outside of school [14]. Parental support is an umbrella term used to represent numerous support behaviors for PA such as encouragement, logistical support, or coactivity [14,15]. This type of parental support is positively correlated with child PA [16,17], with some evidence for stronger effects for girls when they are supported by their mothers rather than by other family members [18,19]. While there has been a growing interest in family-based PA interventions to promote girls’ PA, the evidence for such interventions is mixed [20-22]. These inconclusive findings may be due to factors such as poor study design, small sample sizes, the use of self-report measures, the lack of theory to underpin interventions, the absence of the participant voice in the intervention development process [20,23], and differences between modes of delivery (eg, face-to-face vs eHealth or mobile health [mHealth]) [22,23]. Rapid developments in technology in recent decades have seen an increased use of eHealth and mHealth as modes of delivery for promoting PA in preschoolers [24], children and adolescents [25-27], families [22], and individuals of low SEP [28]. Meta-analyses of eHealth and mHealth PA interventions have reported positive effects for PA-related outcomes in children and adolescents, such as steps per day [25,26] and total PA [26,29], with a lack of improvement in moderate to vigorous PA stated as a limitation [25,29]. Considering the prevalence of smartphone phone use across children, adolescents, and adults [30,31] and the cost-effectiveness, reach, and scalability of mHealth interventions [25,26], there is a pressing need for more robust theory-based mHealth interventions to harness the potential of digital platforms for enhancing PA [22,24,25], particularly for individuals of low SEP [28].

### Intervention Development

There is increasing recognition of the need for guidance to support the robust design of interventions targeting health behaviors such as PA. Specifically, the United Kingdom Medical Research Council (MRC) has developed a framework for complex interventions that provides a systematic process for developing and evaluating interventions across 4 interacting stages [32]. Within this process the importance of using theory, considering context, developing and refining a program theory and related logic model, and engaging with stakeholders is emphasized [32]. Theory offers a valuable organizing framework for the development of effective interventions and is necessary to test hypothesis, identify constructs that effect behavior, and enable study replication and generalization [33,34]. There have been mixed findings reported regarding the effectiveness of interventions that are underpinned by theory [35,36], predominantly explained by a lack of clarity as to how a particular theory’s constructs (ie, mechanisms of action) are targeted and measured within interventions [33,35]. The Behaviour Change Wheel (BCW) builds on MRC guidance and offers a practical guide for how to develop theory- and evidence-based interventions [37]. The BCW is a synthesis of 19 frameworks for classifying behavior change and facilitates the mapping of intervention targets (ie, the behavior, the population, and the context) to specific mechanisms of action (ie, the processes through which behavior change occurs) [38]. At the core of the BCW is the Capability, Opportunity, and Motivation–Behavior (COM-B) model, which proposes that Capability, Opportunity, and Motivation interact to influence behavior. *Capability* refers to the individual’s physical and psychological ability to enact the behavior. *Opportunity* denotes the social and physical resources that facilitate or hinder the behavior. Finally, *Motivation* is defined as the reflective or automatic processes that enable the behavior [37]. The BCW contains 9 different intervention functions that can be applied to target the desired behavior and 7 categories of policy that can be used to deliver these intervention functions. The BCW and associated elements have been successfully used in different contexts to develop interventions promoting PA [39-41]. For example, while using the BCW as part of the development process for a PA app, Truelove et al [39] targeted individuals’ physical and psychological capabilities, physical and social opportunities, and reflective and automatic motivation to increase PA levels in Canadian adults. To achieve this, the intervention functions of education, persuasion, incentivization, training, environmental restructuring and enablement were chosen from the BCW to be included in the app, alongside 2 policy categories (communication and marketing, and environment and social planning) to support the delivery of the intervention functions [39]. One study has used the BCW to develop a mother-daughter PA intervention for adolescent girls [41] by selecting 6 intervention functions (education, persuasion, incentivization, training, modeling, and enablement).

The COM-B components of the BCW can be further understood by using the Theoretical Domains Framework (TDF) [42]. The TDF is a validated integrative framework of 14 theoretical domains synthesized from 128 theoretical constructs and 33 behavioral change theories [42]. Additionally, the TDF presents a comprehensive grouping of the overlapping constructs within behavioral theories and supports the identification and selection of relevant mechanisms of action (eg, knowledge and beliefs about capabilities) for targeting within interventions [37,43]. The TDF has been applied across a variety of settings to inform the development of PA interventions [44,45]. For example, a study by McQuinn et al [45] identified the TDF domains of social influences, environmental context and resources, behavioral regulation, beliefs about capabilities, goals, and reinforcement as target mechanisms of action for a co-designed school-based intervention promoting PA in adolescent girls. However, to date, no intervention has used the TDF to identify mechanisms of action for an intervention promoting PA in preteen girls and maternal PA support behaviors.

An intervention achieves its functions through the use of behavior change techniques (BCTs), which are “the smallest part of the behaviour change intervention content that are that are observable, replicable and on their own have the potential to bring about behavior change” (eg, self-monitoring of behavior and problem-solving) [46]. The Behaviour Change Techniques Ontology (BCTO) offers a reliable and extensive classification system for behavior change intervention content. Using the BCTO is considered best practice, as it contains considerably more BCTs than the original BCT Taxonomy version 1 (BCTTv1), has more precise and clear groupings, labels and definitions, and links to other characteristics of an intervention, such as mechanisms of action [47]. The influence of intervention content (eg, BCTs) on behavior can differ depending on how it is delivered to participants, and therefore vary its effectiveness [48]. The recently developed Behaviour Change Intervention Ontology (BCIO) assists researchers to fully specify and classify intervention characteristics (eg, delivery) in a way that supports improved communication, replication, and implementation of effective interventions [46]. Within the BCIO, the delivery of an intervention is divided into the following components: (1) mode of delivery (ie, the medium through which an intervention is provided) [49], (2) intervention setting (ie, the setting where an intervention takes place) [50], (3) intervention schedule (ie, the timing of intervention components), and (4) intervention style of delivery (ie, the manner in which the intervention is delivered) [48]. Using the BCIO entities to annotate the delivery of an intervention increases our understanding of how the effect of intervention content differs according to the mode and style within which it is delivered [48]. While using the BCIO may be time consuming for researchers, it is increasingly used for evidence synthesis [51,52] and intervention development [53,54]. The BCIO has not yet been applied in PA interventions as it is a new development and is only recently available. To our knowledge, this is the first study to use the BCIO entities to annotate the characteristics of an intervention targeting PA in children.

### Objectives

Alongside this increased emphasis on a systematic theory-informed intervention development, a collaborative approach to intervention design involving the end users of research is essential. Co-design methods ensure meaningful involvement of the end user in the research process [55] by enabling the specific needs and preferences of the target population to be recognized and allowing for the identification of potential implementation challenges early in the intervention development process [56]. Indeed, research that involves end users in the design process leads to interventions that are more contextually relevant and thus more effective [57]. However, while there is a continued call for greater involvement of young people in the research process through participatory methods such as co-design [58,59], only a few studies on family-based interventions targeting girls’ PA [41] or on PA in teenage girls from lower SEP have applied such methods [45,60]. Therefore, the purpose of this study was to provide a detailed outline of the systematic process undertaken to using the BCW and TDF to develop an evidence-based and theoretically informed behavior change intervention, using co-design methods, to promote PA in preteen girls incorporating maternal support behaviors, before preliminary testing for feasibility and efficacy.

## Methods

### Overview

**Figure 1:**
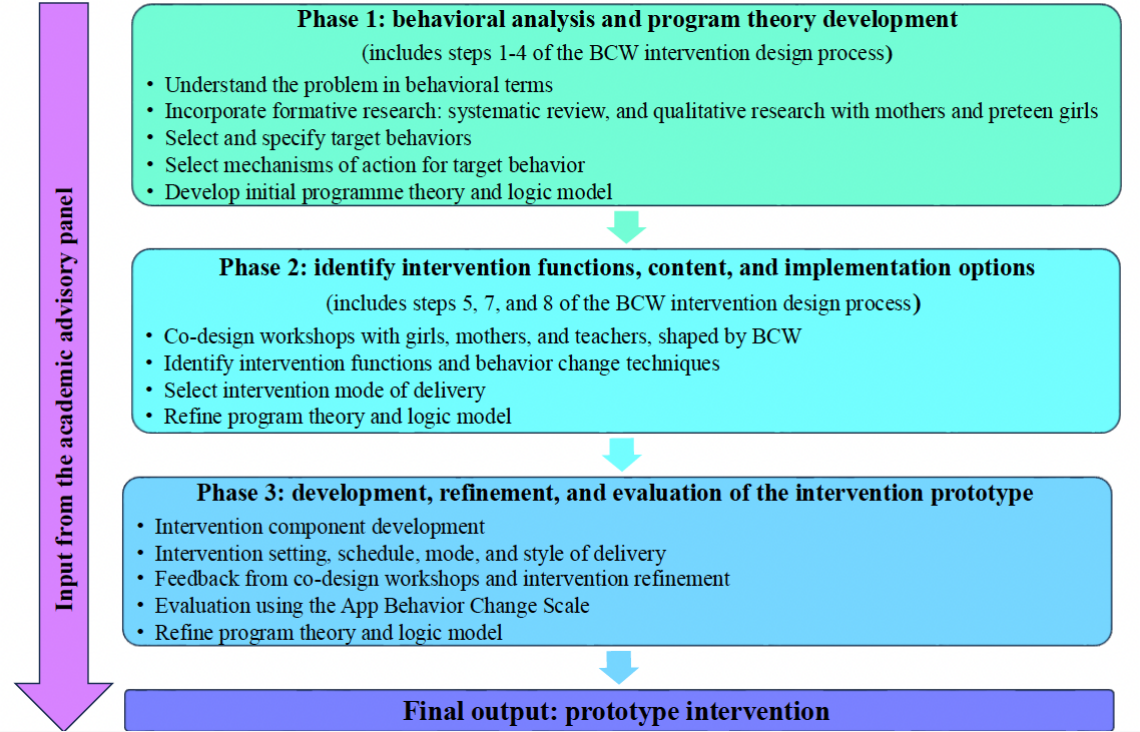
Overview of the intervention development process. BCW: Behaviour Change Wheel.

This study was informed by the initial development stage of the MRC framework for complex interventions [32]. In line with MRC guidelines, a program theory and logic model were developed and refined throughout the intervention development process. A program theory is a tool that can be used to unpack the relationship between the intervention activities and intended outcomes [61]. Logic models can assist in visually representing the program theory to effectively communicate with research team members and stakeholders [61]. The intervention prototype developed across 3 phases (Figure 1). Phase 1 was guided by the steps in the BCW process [37] and also incorporated the TDF to identify more specific mechanisms of action [42]. Phase 2 involved co-design workshops with stakeholders (ie, mothers, preteen girls, and primary school teachers) to identify potential intervention components and mode of delivery. In phase 3, the prototype was refined through an iterative and dynamic process based on evidence, theory, and input from additional co-design workshops with stakeholders (ie, mothers, preteen girls, and primary school teachers). An academic advisory panel provided guidance throughout the process. The BCIO entities were annotated to report the intervention characteristics; some of the BCIO unique identifiers are provided in the manuscript, with a full list available in Multimedia Appendix 1.

### Ethical Considerations

Ethics approval was obtained by the University College Dublin’s Human Research Ethics Committee (LS-22-62) before study commencement. No payments or incentives were offered for participation. Information packs containing information sheets and consent and assent forms were distributed to mothers, children, and teachers alike.

### Recruitment

#### Co-Design Participants

A suburban primary school identified by the Department of Education’s Delivering Equality of Opportunity in Schools (DEIS) program was identified as suitable for this study. The Department of Education uses the DEIS classification system to support students attending schools situated in communities at risk of social and economic disadvantage [62]. To classify schools as meeting the DEIS criteria, data from the Department of Education’s online database and the HP Deprivation Index for Small Areas (HP Index) are used [62]. The HP Index is a process that measures the relative affluence or disadvantage of small geographical areas using categories such as demographic growth, dependency ratios, education levels, single parent rate, overcrowding, social class, occupation, and unemployment rates [62]. The school in this study is a mixed primary school based in a suburban town located 10 km from Dublin city center, Ireland, with approximately 520 pupils and 33 teachers. After discussions between the lead author (CB) and the school principal, the school principal invited mothers and female guardians of girls aged 10 to 12 years, girls aged 10 to 12 years, and teachers to take part in the study. All girls who were aged between 10 and 12 years and from fourth, fifth, or sixth class were eligible to take part.

#### Academic Advisory Panel

As part of the research process, an academic advisory panel was established to discuss the findings from the co-design workshops, the use of theory, and support the research team (CB and JM). This panel consisted of 3 academics (GO’D, AK, and RER) with expertise in PA, sedentary behavior, and the development of complex interventions and co-design methodologies. Both GO’D and AK are experienced qualitative researchers and have conducted previous studies exploring PA and sedentary behavior using the TDF, and RER is an experienced researcher with an applied focus on PA during life transitions.

### Intervention Prototype Development Process

#### Phase 1: Behavioral Analysis and Program Theory Development

Phase 1 included steps 1 to 4 of the BCW intervention design process [37]. In line with this approach, the definition of the problem in behavioral terms (step 1) was based on findings from previous literature (ie, preteen girls are not active enough) [1,4]. A systematic review of mother-daughter interventions and formative qualitative research (ie, interviews with 29 mothers of preteen girls and 19 focus groups with 107 low-SEP preteen girls) was then conducted to further understand the problem and the related factors. This was followed by selection and specification of the intervention target behaviors (steps 2 and 3 of the BCW process). After these steps, CB and JM used the TDF to identify the barriers and enablers of the target behaviors. These were presented to the academic advisory panel (GO’D, AK, and RER) to establish what needs to change to achieve the target behaviors (step 4; Multimedia Appendix 2 [6,14,63-76]). This led to the selection of specific mechanisms of action to be targeted within the proposed intervention. An initial program theory and related logic model for the intervention were then developed.

#### Phase 2: Identify Intervention Functions, Content, and Implementation Options

This phase includes steps 5, 7, and 8 of the BCW intervention design process [37]. Three co-design workshops took place at the school premises during school hours and were facilitated by CB and JM. Three separate groups took part in a co-design workshop: (1) mothers of preteen daughters (n=9), (2) preteen girls (n=10), and (3) teachers (n=6). The workshops took place in April 2023, with a mean duration of 52 (SD 1.9) minutes. The aim of these workshops was to identify potential intervention functions, BCTs, and modes of delivery to target the proposed mechanisms of action identified in phase 1. A range of age-appropriate and interactive methods were used in these co-design sessions (Multimedia Appendix 3). For example, to encourage participants to think about the practical application of their suggestions to a wide variety of mothers and girls, personas of mothers and girls who were individually, socially, and geographically diverse were provided [77]. Using the information gathered from these co-design sessions, along with findings from phase 1 and further consultation with the academic advisory panel (GO’D, AK, and RER), intervention functions (step 5), BCTs (step 6), and a proposed mode of delivery (step 8) were selected by the research team (CB and JM). The program theory and logic model were also refined.

#### Phase 3: Development, Refinement, and Evaluation of the Intervention Prototype

This phase involved incorporating the findings from phase 2 into the development of the intervention components. A second series of co-design workshops (n=3) took place in the school premises during school hours and were facilitated by CB and JM. The same participants as phase 1 took part. The three separate workshop groups were (1) mothers of preteen daughters (n=6), (2) preteen girls (n=10), and (3) teachers (n=3). The workshops took place in June 2023, with a mean duration of 44 (SD 5) minutes. The aim of the workshops was to obtain participants’ feedback on the acceptability of the proposed intervention components (Multimedia Appendix 4). Following these workshops, the research team (CB and JM) discussed the findings from the workshops, the proposed intervention components, and the use of theory with the academic advisory panel (GO’D, AK, and RER). The 21-item App Behavior Change Scale [78] was also used by the research team to ensure that relevant behavior change components were appropriately included. This scale has been used in several studies targeting PA to assess intervention effectiveness [39,79]. The program theory and logic model were refined for the final time.

## Results

### Phase 1: Behavioral Analysis and Program Theory Development

As described in the Introduction section, the identified problem behavior was the decline in PA as children transition to adolescence, with this decrease in activity levels particularly prevalent for girls of lower SEP [1,4]. Children whose parents support PA are likely to have higher overall levels of activity than children whose parents do not support their PA, with stronger effects when that support is provided by a parent of the same gender [80,81]. The formative research related to this study is described in previous studies, [82-84] therefore a brief description of it is provided here. A review of behavior change theories and techniques used in mother and daughter PA interventions highlighted a lack of clarity as to why interventions were effective or not and the increased need for a stronger theoretical basis for future interventions as well as enhanced reporting of how these interventions are developed [82]. Qualitative formative work with mothers of preteen girls highlighted barriers and enablers related to engaging in PA-supportive behaviors with their daughters [83]. These ranged from individual-level factors such as their PA-related identity and their confidence to engage in supportive behaviors to social and environmental factors such as the role of other family members and the infrastructure within their communities and their daughters’ schools [83]. Finally, qualitative work was conducted with preteen girls who discussed barriers and enablers to their PA, such as the importance of skills and confidence to support their engagement in PA and strengthen their self-identity for PA alongside the important role of family members, friends, teachers, and coaches [84]. On the basis of this formative work, 2 related behaviors were deemed appropriate to target as part of the intervention. The first behavior was to improve mothers’ support for their preteen daughters’ PA and, in doing so, indirectly increase the likelihood of preteen girls engaging in PA. The second behavior being targeted was to increase preteen girls’ PA. These behaviors are presented in Table 1 in terms of who needs to perform the behavior, when, where, and with whom.

The academic advisory panel then reviewed the analysis of the barriers and enablers to the target behaviors. Following discussion with the advisory panel, the research team then chose 11 of the 14 TDF domains as proposed mechanisms of action for enabling these target behaviors (Table 2).

**Table 1:** Specification of target behaviors of interest.

	Target behavior 1	Target behavior 2
What behavior	Improve mothers’ PA^a^ support behaviors (eg, encouragement, logistical support, coactivity, and environmental and regulatory support) for their preteen daughters	Increase preteen daughters’ PA (includes active travel, sport, family activities [bike rides and walks], and outdoor play)
Who	Mothers of preteen daughters of low SEP^b^	Preteen daughters of low SEP
When	Daily	Daily
Where	In their household residence (BCIO^c^: 026009), sport and exercise facility (BCIO: 026030), and outdoor environment (BCIO: 026044)	In their household residence (BCIO: 026009), sport and exercise facility (BCIO: 026030), and outdoor environment (BCIO: 026044)
With whom	Preteen daughters	Friends, mothers, and other family members

^a^PA: physical activity.

^b^SEP: socioeconomic position.

^c^BCIO: Behaviour Change Intervention Ontology.

**Table 2:** Mechanisms of action, intervention functions and behaviour change techniques for mother-daughter intervention.

Mechanisms of action and what needs to happen for behavior change to occur	Intervention functions for improving maternal PA^a^ support and promoting PA in preteen girls	BCTs^b^ from BCTO^c^ for improving maternal PA support and promoting PA in preteen girls
*Knowledge* Develop mothers’ and daughters’ understanding of the following: The rationale and purpose of the program The types and benefits of PA and PA guidelines How to be physically active The types and benefits of maternal PA support behaviors How to perform maternal PA support Typical challenges experienced by mothers while engaging in PA support (eg, pushback from daughter) Typical challenges experienced by preteen girls while engaging in PA support (eg, friends not active) Available resources to facilitate engagement in PA and PA support	*Education* About the rationale and purpose of the program About ways of enacting desired behavior and avoiding undesirable ones Provide credible, appealing information that can be used to enact target behavior Provide clear, consistent, and standardized messages about maternal PA support and PA Provide information to address prevalent misconceptions about maternal PA support and PA behaviors	Instruct how to perform behavior BCT (BCIOd:007058) Inform about health consequences BCT (BCIO:007063) Inform about social consequences BCT (BCIO:007064) Inform about environmental consequences BCT (BCIO:007176) Present information from credible influence BCT (BCIO:007075)
*Skills* Develop skills to do the following: Select and engage in PA and PA-supportive behaviors Apply problem-solving and set and review personalized goals for PA and PA support behaviors Monitor progress of physical activity behaviors Monitor progress in supporting daughter to be active; overcome the challenges encountered while engaging in selected PA and PA support behaviors	*Training* Practice and engage in PA support and PA behaviors Engage in problem-solving and select and review goals related to target behavior Monitor progress when engaging in PA support and PA behaviors Engage in behavioral strategies to overcome challenges associated with providing support or being active	Goal strategizing BCT (BCIO:007008) Provide feedback on behavior BCT (BCIO:007023) Self-monitor of behavior BCT (BCIO:007024) Instruct how to perform behavior BCT (BCIO:007058) Demonstrate the behavior BCT (BCIO:007055) Practice behavior BCT (BCIO:007094) Context-specific repetition of behavior BCT (BCIO:007096) Set graded tasks BCT (BCIO:007100)
*Social role and identity* Develop mothers’ identity as a person who provides support for their daughters’ PA Develop mothers’ and daughters’ identity as a person who is physically active	*Education* About how to link supportive and PA behaviors to other intrinsic goals Provide information about positive experiences when supporting their daughter to be active or being active and how to overcome associated challenges Provide information about extra resources available to help mothers provide support or preteen daughters be active when program ends *Persuasion* Highlight compatibility with current identity, but expand it to include maternal PA identity or PA behaviors and social identities Emphasize the role of mother as change agent for daughter and in family	Social support BCT (BCIO:007028) Inform about social consequences BCT (BCIO:007064) Prompt social comparison BCT (BCIO:007073) Practice behavior BCT (BCIO:007094) Present information from credible influence BCT (BCIO:007075) Identify self as role model BCT (BCIO:007158) Reframe past behavior BCT (BCIO:007056) Adopt changed self-identity BCT (BCIO:007160)
*Beliefs about capabilities* Improve perceived competence in ability to do the following: Perform selected PA and PA support behaviors Use problem-solving, goal setting, and action planning to engage in PA and PA support Ability to monitor progress Overcome challenges encountered while enacting PA and PA support Engage in long-term PA and PA support behaviors	*Persuasion* Enhance perceived competence to problem solve, actions plan, select and monitor goals, and self-monitor PA support and PA behaviors Encourage mothers to believe that providing support is possible or daughters to believe that being active is possible, even given constraints of their circumstances *Enablement* Assist mothers or daughters in problem-solving and action planning to overcome barriers to providing support or being active *Modeling* Present real-life examples of mothers or preteen girls in similar circumstances	Goal strategizing BCT (BCIO:007008) Social support BCT (BCIO:007028) Instruct how to perform behavior BCT (BCIO:007058) Demonstrate the behavior BCT (BCIO:007055) Practice behavior BCT (BCIO:007055) Set graded tasks BCT (BCIO:007100) Advise how to reduce negative emotions BCT (BCIO:050344) Persuade about personal capability (BCIO:007137) Prompt focus on past success BCT (BCIO:007139) Prompt self-talk BCT (BCIO:007140)
*Beliefs about consequences* Enhance mothers’ expectations related to the positive consequences of engaging in selected PA support Enhance mothers’ and daughters’ expectations related to the positive consequences of engaging in selected PA behaviors	*Education* Explore beliefs and attitudes related to PA and the associated health benefits Explore beliefs and attitudes between providing PA support and expected outcomes *Persuasion* Enhance beliefs that being physically active has positive health benefits in the short and long term Provide expert information about how, where, and why to be active Enhance beliefs that providing support for daughters’ PA would be beneficial Provide expert information about the short- and long-term benefits of providing PA support *Modeling* Provide demonstrations of mothers of teen girls or preteen girls to show the benefits they received as a result of providing support or being active	Inform about health consequences BCT (BCIO:007063) Inform about social consequences BCT (BCIO:007064) Inform about environmental consequences BCT (BCIO:007176) Monitor emotional consequences BCT (BCIO:007066) Demonstrate the behavior BCT (BCIO:007055) Prompt social comparison BCT (BCIO:007073) Present information from credible influence BCT (BCIO:007075)
*Intentions* Increase mothers’ and daughters’ autonomous motivation to Engage in and maintain selected PA or PA support behaviors Engage in problem-solving and setting and reviewing goals to facilitate engagement in selected PA and PA support behavior Engage with tools to monitor progress	*Education* Inform about importance of formulating intentions of how and where to provide support or be active *Persuasion* Encourage mothers and daughters to consider why being active might be important to them and the benefits they will receive Encourage mothers to consider why providing PA support may be important to them and how it would benefit their daughter and other family members *Modeling* Provide demonstrations of mothers and preteen girls describing their experiences of setting short- and long-term intentions to be provide support or be active and the associated benefits	Set behavior goal BCT (BCIO:007003) Inform about health consequences BCT (BCIO:007063) Inform about social consequences BCT (BCIO:007064) Inform about environmental consequences BCT (BCIO:007176) Instruct how to perform behavior BCT (BCIO:007058) Demonstrate the behavior BCT (BCIO:007055) Prompt social comparison BCT (BCIO:007073) Present information from credible influence BCT (BCIO:007075)
*Goals* Support mothers and daughters to Use action planning, problem-solving, and goal setting to facilitate engagement in selected PA and PA support behaviors Use tools to monitor progress Overcome challenges encountered while setting and reviewing goals	*Training* Select and review personalized goals related to the target behavior *Enablement* Provide support and guidance for setting realistic goals for mothers to provide support or to preteen girls to be physically active Affirm small achievable and interim goals and successes Prompt planning to provide PA support or be active during and after the intervention	Set behavior goal BCT (BCIO:007003) Goal strategizing BCT (BCIO:007008) Action planning BCT (BCIO:007010) Provide feedback on behavior BCT (BCIO:007023) Self-monitor behavior BCT (BCIO:007024) Instruct how to perform behavior BCT (BCIO:007058) Demonstrate the behavior BCT (BCIO:007055) Practice behavior BCT (BCIO:007055) Set graded tasks BCT (BCIO:007100)
*Environmental context and resources* Provide knowledge of and access to a variety of PA opportunities available so that mothers can support daughters’ PA and daughters can engage in PA Provide materials or equipment so that mothers can support their daughters PA or daughters can be active	*Environmental restructuring* Provide mothers or preteen daughters with practical equipment that can enable them to be active, for example, skipping ropes and balls Provide mothers and daughters with access to feasible and realistic options that enable them to be active during and after the intervention *Enablement* Provide practical support for mothers and daughters to action plan and problem solve to engage in PA support and PA behaviors	Goal strategizing BCT (BCIO:007008) Action planning BCT (BCIO:007010) Social support BCT (BCIO:007028) Present information from credible influence BCT (BCIO:007075) Add objects to the environment BCT (BCIO:007156)
*Social influences* Develop mothers’ and daughters’ understanding of the type of support available to them regarding supporting their daughters’ PA and being active Develop mothers’ and daughters’ ability to engage with the social support available to them	*Modeling* Provide demonstrations of other mothers and girls describing their experiences for seeking and receiving social support and the benefits they received as a result *Enablement* Prompt mothers and preteen daughters to seek social support and provide examples of types of social support available to them	Social support BCT (BCIO:007028) Advise to seek instrumental support BCT (BCIO:007030) Demonstrate the behavior BCT (BCIO:007055) Prompt social comparison BCT (BCIO:007073) Present information from credible influence BCT (BCIO:007075) Provide positive social consequence for behavior BCT (BCIO:007265) Persuade about personal capability BCT (BCIO:007137)
*Emotion* Promote positive and reduce unpleasant emotions associated with providing PA support (eg, embarrassment while being active with daughter) and being active (eg, enjoyment in activities)	*Persuasion* Help mothers and daughters recognize the positive feelings associated with providing support or being active *Enablement* Provide safe and nonjudgmental environment for mothers or daughters to explore emotions around providing support or being active Provide opportunities for mothers or daughters to evaluate their emotional state after providing support or being active	Goal strategizing BCT (BCIO:007008) Social support BCT (BCIO:007028) Monitor emotional consequences BCT (BCIO:007066) Present information from credible influence BCT (BCIO:007075) Advise how to reduce negative emotions BCT (BCIO:050344) Reframe past behavior BCT (BCIO:007056)
*Behavioral regulation* Develop mothers’ and daughters’ ability to do the following: Select and apply PA or PA support behaviors into their daily life Implement tools to monitor PA or PA support progress	*Training* Provide means so that mothers and daughters can assess their progress during the intervention and in the future *Enablement* Provide opportunity, support, and tools to self-monitor PA support or PA behaviors and related habits	Goal strategizing BCT (BCIO:007008) Action planning BCT (BCIO:007010) Self-monitor behavior BCT (BCIO:007024) Instruct how to perform behavior BCT (BCIO:007058) Demonstrate the behavior BCT (BCIO:007055) Practice behavior BCT (BCIO:007055) Substitute behavior BCT (BCIO:007095) Context-specific repetition of behavior BCT (BCIO:007096) Provide positive social consequence for behavior BCT (BCIO:007265) Advise how to reduce negative emotions BCT (BCIO:050344) Prompt self-talk BCT (BCIO:007140)

### Phase 2: Identify Intervention Functions, Content, and Implementation Options

The co-design workshops led to a number of recommendations from preteen girls, mothers, and teachers. These recommendations are illustrated in Table 3 using exemplar quotes and were categorized under a range of intervention functions as per the BCW intervention design process [37]. The intervention functions included education, training, persuasion, modeling, enablement, incentivization, and environmental restructuring. Potential modes of delivery discussed included face-to-face delivery, remote synchronous delivery (eg, Zoom Communications, Inc), or the use of a mHealth application. The mothers’ group recommended the use of a mHealth application as a potential mode of delivery. These recommendations informed the selection of potential intervention functions, BCTs, and a proposed mode of delivery (ie, mHealth application) for each target behavior by the research team and were presented to the academic advisory panel for review. The final listing of intervention functions and BCTs for each mechanism of action are presented in Table 2. The academic advisory panel suggested applying of the principles of self-determination theory (SDT) [85] to the mHealth application content to enhance the communication style within which it is delivered [48].

**Table 3:** Summary table of the co-design workshops.

	Summary of the recommendations from workshops	Example quotes	Related intervention functions
Improving mothers’ knowledge and understanding of PA^a^ and PA support	Provide mothers with information about the different types of PA, the benefits of PA, how to be active, and how much PA is recommended for daughters and themselves Provide mothers with information and instruction about how to support their daughters, particularly as they transition into teenage years Provide mothers with more information about what is available to them in their local area for their daughters to be active or for them to be active with their daughters Information could be provided through videos, social media, websites, an app, parent-teachers association, and word of mouth	“Maybe something about their mental and physical development, psychosocial development at this age that would help mothers understand what they’re going through.” [Imelda, daughter in fifth class] “Making sure mothers know how often children, girls at that age should be exercising each week. Maybe if they’re not conscious that they’re doing their weekly exercise, how are they supposed to pass it on to their children.” [Kate, primary school teacher] “if you were thinking of an app and giving people ideas, could add the likes of yoga and stuff.” [Susan, daughter in sixth class]	Education Training
Persuading mothers to support their daughters to be active	Present mothers with examples of other mothers, coaches, teachers, and local sports partnership representatives explaining how, where, and the benefits of supporting daughters to be active Provide examples of other mothers supporting their daughters to be active, how they overcame challenges, and being active themselves or with their daughters or family members Use mainstream media, social media, videos, websites, apps, and word of mouth to promote positive messages	“Or other mammies probably. I think other mammies would be good to see. Well, if they can balance it, I’m sure there’s ways around that we can balance it.” [Niamh, daughter in fourth class] “Someone giving them their personal story” [Sharon, primary school teacher] “an app would be great because the kids are on it...you can challenge your friends or family members and track what you have done.” [Sinead, daughter in sixth class]	Persuasion Modeling
Practical help for mothers to engage in support behaviors	Assistance with cost of activities, thus provide opportunities to try out in school or community for free first Provide materials for mothers to plan, record, and monitor their PA support behaviors at their own time and pace Feedback on behavior, in particular, if they engage in activities with daughters, either through technology or in person	“And the cost of living at the moment is crazy as well. Like you should be really dropping. If the cost of it came down a bit, I think a lot more people would do it, absolutely.” [Michelle, daughter in fourth class] “If you’re doing that at your own pace, in your own time. There was ideas on of, you can do this today, tomorrow, next week, or whatever, but also a blank space that you could fill in what you’ve done. I wasn’t able to do this, but I did this, or we done that.” [Sinead, daughter in sixth class] “You can break it into profiles like you can have yours, your partners, your daughters, whatever. It’s under the one branch, basically. But you have your own little sections as well, where there’s probably things tailor-made for you for that age group. You put in your age, you put in your interest or something...Like Netflix.” [Imelda, daughter in fifth class]	Incentivization Environmental restructuring
Social support for mothers	Provide opportunities for mothers to get support from other mothers of preteen daughters, for example, Facebook group Opportunities to get to know other mothers at daughters’ activities	“every club should have a mammies group.” [Niamh, daughter in fourth class] “then you could have like a chat group for them (mothers) on the app.” [Susan, daughter in sixth class] “Yeah, support network through your app, through your group or whatever. Just like, oh, I’ve done this week, you might like it or I found this video, you might like it whatever.” [Anna, primary school teacher] “Because some of the other parents have groups themselves where they can keep in contact. If one parent doesn’t want to do it, the second parent may motivate them to do it. While we’re all doing this together.” [Joe, primary school teacher]	Enablement
Improving daughters’ knowledge and understanding of PA	Provide daughters with information and instructions about the different types of PA, the benefits of PA, how to be active in their leisure time, and how much PA is recommended Provide daughters with information about what is available to them in their local area to be active. Information could be provided through videos, social media, websites, an app, and word of mouth	“tips on how to play a game or rules of the game.” [Emily, fifth class] “we could have like speakers, people going into the classroom and talking to girls to join sports.” [Sophie, sixth class] “Some people post on YouTube how to do skills. If you’re a beginner and you want to learn some skills, you could just look at some YouTube videos and then that would do it.” [Robyn, sixth class] “But the danger is that there are parents who don’t know and don’t care, and they probably won’t look at an app. I would be afraid of that happening...let the child have their own profile.” [Jennifer, daughter in sixth class]	Education Training
Persuading daughters to be active	Provide encouragement for daughters with examples of coaches, teachers, and local sports partnership representatives explaining how, where, and the benefits of being active Provide examples of other preteen girls and older adolescent girls being active and how they overcame challenges Use mainstream media, social media, videos, websites, apps, and word of mouth to promote positive messages	“You could tell them how worth it will be when they get stronger and healthier and they can run more. Basically, classes, carrying, and shopping, you’ll just get quicker and it’ll become easier.” [Emily, fifth class] “They might because they are their own age feel like they are like them.” [Aisling, fifth class] “Probably get girls that do sports, like making ads or something. If girls are watching their phones and you could do that.” [Evie, sixth class] “Like that, if there were videos that they (girls) could click into.” [Sharon, primary school teacher]	Persuasion Modeling
Practical help for daughters to engage in leisure time PA	Cost of activities is a barrier, so provide opportunities to try them out in school or community for free first (initial cost) Provide materials for daughters to plan, record, and monitor their PA behaviors, including opportunity to arrange a reward of their choice for themselves Feedback on behavior if they engage in activities Provide equipment and merchandise for daughters to practice with at home, for example, footballs, basketballs, skipping ropes, T-shirts, jerseys, hoodies, water bottles, and merchandise	“You can write a to-do list, so that way you can be more motivated to keep on schedule.”[Emily fifth class] “They could lend them a ball to practice.” [Maisie, fifth class] “Something like if you have all the jumpers and the jersey’s, it makes you feel a part of the team, so you want to go again because you are part of this team.” [Evie, sixth class] “If you could practice it at home to see if you like it.” [Sarah fourth class]	Incentivization Environmental restructuring
Social support for daughters	Provide opportunities for daughters to get support from other preteen girls, for example, bringing friends to activities and opportunities to ask girls their own age about certain activities Opportunities to get to know other girls at activities. Can be organized by coaches, teachers, and other club members	“They could get a friend to join with them so that they have someone to talk to.” [Aisling, fifth class] “Maybe if one of your friends was not on the team can now join the sport, you could even ask them if they wanted to come down to maybe one of your matches so they could have a look and see what the sport is all about and that might make them want to join.” [Sophie sixth class]	Enablement

^a^PA: physical activity.

### Phase 3: Development, Refinement, and Evaluation of the Intervention Prototype

#### Intervention Component Development

The research team developed separate mobile apps for each target behavior (ie, mothers’ support behaviors and preteen daughters’ PA) using the *Pathverse* app design platform for mHealth research [86]. This platform enables researchers to develop mobile apps for testing without the requirement of software developers. It is a “no-code” development platform, which allows researchers to create a mobile app with “drag and drop” features instead of coding [87]. The *Pathverse* platform includes features such as the design of customized multimedia content, implementation of participant surveys, provision of self-monitoring tools, setting of personalized goals, the customization of app notifications, digital badges, and a community group chat option [86]. Intervention components were developed within this platform to ensure that the relevant mechanisms of action were targeted and the related BCTs were enacted. Examples of how the intervention components relate to the targeted mechanisms of action are provided in Tables 4 and 5 for mothers and preteen daughters, respectively. For example, for the mothers’ intervention, app module 3 titled “What does supporting your daughter involve?” includes infographics about the benefits of and the different ways for mothers to support their daughter to be active. It also includes videos of mothers describing their experiences of engaging in different supportive behaviors. Similarly, in the daughters’ intervention, app module 3 titled “Why should you be active?” includes infographics and a video about the benefits of being active as well as a video of a preteen girl describing her experiences of engaging in PA. The mechanisms of action that these modules target are “knowledge,” “beliefs about consequences,” and “social influences.”

**Table 4:** Intervention components mapped to the mechanisms of action in the mothers’ intervention.

	Intervention components, activities, and resources	Mechanisms of action
Week 1: introduction to the study and group meeting (included after feedback from the co-design workshops, session 2)	Face-to-face meeting with the mothers who are taking part in the study and introducing them to the research team and providing information about the study and consent forms, equipment and merchandise that are part of study (eg, footballs, skipping rope, yoga mat, T-shirts, and water bottles), demonstration of how to download the app and navigate the modules and features of the app, and inform mothers that they will receive certificate for taking part at the end of the study.	Knowledge Beliefs about consequences Environmental context and resources
App module 1: getting started	Includes a video with a welcome message and brief description about the study; a video demonstrating how to use the app and answer survey questions; and a survey with questions about demographics, PAa levels, and providing support for daughter’s activities	Knowledge
App module 2: what is PA and why is it important?	Includes infographics about the module objectives, PA and benefits of being active, how active adults should be, how active children and teenagers should be, the benefits of PA for children and adolescents, and how active our teenage girls currently are A multiple-choice challenge question about how active children should be and a digital badge of congratulations for reaching the end of the module	Knowledge Beliefs about consequences
App module 3: what does supporting your daughter to be active involve?	Includes infographics about the module objectives; why mothers were chosen for the study; the benefits of and ways to support their daughter to be active, for example, providing transportation to their daughter’s activities; spectating at daughter’s activities; and how and where mothers can be active with their daughters A video with a mother of preteen girls describing her experience of spectating at her daughter’s activities A multiple-choice challenge question about what a mother can do to help support their daughter to be active and a digital badge of congratulations for reaching the end of the module	Knowledge Beliefs about consequences Social influences
App module 4: who can help you support your daughter?	Infographics about the module objectives, the benefits of social support, having families as a source of support, how friends can support, how neighbors and people in the local community can support, how a daughter’s friends can facilitate providing support, how coaches and teachers can support, and how support groups both web-based and in the local community can help mothers A video of mothers of preteen girls describing how they avail the social support available to them A multiple-choice question about who can help mothers with the day-to-day challenges of supporting their daughter to be active and a digital badge of congratulations for reaching the end of the module	Knowledge Beliefs about consequences Intentions Social influences
App module 5: tips to help you support your daughter	Infographics about the module objectives, the challenges mothers face when supporting their daughter and how to overcome them, to remember why supporting their daughter can help do what is important to them, tips for how to get started when feeling overwhelmed, details about shared decision-making and how it could be helpful, and how to manage a lapse in behavior A video with tips and advice from a role model, for example, mother of an athlete and how they support their daughter A multiple-choice questions about the types of support messages mothers would like to receive throughout the study as push notifications (eg, reminders, encouragement, praise, affirmations, or inspirational) and a digital badge of congratulations for reaching the end of the module	Knowledge Skills Identity Beliefs about capabilities Beliefs about consequences Intentions Social influences Emotion
App module 6: next steps: planning to support your daughter	Includes infographics about the module objectives, what is goal setting, why and how to set goals, challenges associated with goal setting and how to overcome them, and showing mothers how they can self-monitor their progress in the app Provide selection goals related to maternal PA support (eg, spectate at daughter’s activity and mother and daughter coactivity) Mothers select and set a PA support goal of their choice, including when and where and how often the support behavior would be enacted, and a digital badge wishing mothers “good luck” with their chosen goal	Knowledge Skills Beliefs about capabilities Beliefs about consequences Intentions Goals Behavioral regulation
App module 7: booster module (included after feedback from co-design workshops, session 2)	Includes infographics about the module objectives; recap on maternal PA support behaviors; how to overcome potential barriers to providing support, for example, using if...then statements; and how lapses in behaviors are normal A video with messages of encouragement and support from other mothers of teenage girls and a digital badge of congratulations for reaching the end of the module	Knowledge Identity Beliefs about capabilities Beliefs about consequences Intentions Social influences Emotion Behavioral regulation
App module 8: final module	Includes infographics with a summary of the study, recap on maternal PA support behaviors, goal setting, how to overcome challenges, where to look for social support, and what to do next A survey with questions about PA levels for mothers and daughters and regarding providing support for daughter’s activities A survey providing feedback about acceptability and feasibility of the study and a digital badge of congratulations for reaching the end of the module	Knowledge Beliefs about consequences Intentions Social influences Behavioral regulation
App icon: resources	Links to external websites providing information on local resources, family support services, and community events. Includes podcasts about parenting for PA with a focus on mothers and girls, videos of skills or activities that mothers can practice with daughter (eg, yoga, exercises, and football skills), and videos of other mothers of preteen girls discussing and sharing their experiences	Knowledge Skills Beliefs about capabilities Beliefs about consequences Intentions Social influences Emotion
App icon: goals	Summary of goals set for the duration of the study Mothers can review progress of goals set in the module, for example, spectate at daughter’s activity or walk to school with daughter twice a week. Mothers can manually set and record additional goals of their choice	Intentions Goals Behavioral regulation
App icon: trackers history	Option available for mothers to self-monitor their progress of goals set that are paired with a smartwatch that is synced with the app, for example, step count and exercise minutes. Mothers can self-monitor their daily steps and exercise minutes manually. Mothers manually self-monitor an activity (eg, walked to school with daughter), rate their enjoyment factor, and record any notes or points of interest	Intentions Goals Behavioral regulation
App icon: chat	A feature that enables mothers to join a community forum to send and receive messages to and from the research team; an interactive forum where mothers share their experiences and strategies with other mothers who are partaking in the study; and avail of opportunities to meet other mother and daughter participants for group activities, as per information provided by the research team or as suggested by other participants	Social influences Emotion
Intervention feature: motivational messages	Tailored prompts or cues sent as push notifications to mothers that are relative to their chosen goals and generic messages of encouragement, praise, or inspiration that change each day	Beliefs about capabilities Identity Social influences Emotion
Week 8: conclusion of study and group meeting (included after feedback from co-design workshops, session 2)	Face-to-face group meeting with mothers to award mothers with a certificate of completion, receive feedback on the intervention, and answer any questions	Social influences

^a^PA: physical activity.

**Table 5:** Intervention components mapped to the mechanisms of action in the preteen girls’ intervention.

	Intervention components, activities, and resources	Mechanisms of action
Week 1: introduction to the study and group meeting (included after feedback from the co-design workshops, session 2)	Face-to-face meeting with the girls who are taking part in the study and introducing them to the research team and providing information about the study and consent forms, equipment and merchandise that are part of study (eg, footballs, skipping rope, yoga mat, T-shirts, and water bottles), demonstration how to download the app and navigate the modules and features of the app, and answers to queries or concerns girls may have Inform girls that they will receive a certificate for taking part at the end of the study	Knowledge Beliefs about consequences Environmental context and resources
App module 1: welcome to our study	Includes a video with a welcome message and a brief description about the study; a video demonstrating how to use the app and answer survey questions; a survey with questions about demographics, PAa levels, and being active, and a digital badge of congratulations for reaching the end of the module	Knowledge
App module 2: what is PA?	Includes infographics about the module contents, what is PA, how active preteen girls need to be, and different ways to be active A multiple-choice challenge question about how many minutes per day should preteen girls be active for and a digital badge of congratulations for reaching the end of the module	Knowledge Beliefs about consequences
App module 3: why should you be active?	Infographics about the module objectives and the benefits of being active Videos about the benefits of being active, with a preteen girl describing her experiences of being active and the associated benefits A multiple-choice challenge question about the benefits of being physically active and a digital badge of congratulations for reaching the end of the module	Knowledge Beliefs about consequences Social influences
App module 4: how can you be active?	Includes infographics about the module objectives; walking or wheeling to school; outdoor play in the neighborhood with friends; going to local parks, woods, and playgrounds with family members; walking the dog as a way to be active; and yoga as a way to be active Videos about ways in which preteen girls can be active, of interview with famous female sports stars describing their experiences of playing sport and being active, with mothers and daughters dancing together as a way to be active, and of exercises that can be implemented at home as a way to be active A question about favorite way to be active and a digital badge of congratulations for reaching the end of the module	Knowledge Skills Beliefs about consequences Intentions Social influences
App module 5: who can you be active with?	Includes infographics about the module objectives, who can support girls to be active and the benefits of social support, having fun with friends at school, playing with children in the neighborhood, and being active with family members Video of other preteen girls sharing their experiences of who they are active with A multiple-choice question about who girls can be active with and a digital badge of congratulations for reaching the end of the module	Knowledge Beliefs about consequences Intentions Social influences
App module 6: tips to help you be active	Includes infographics about the module’s objectives; why some girls do not want to be active; about how family members or friends can support you to be active; and remembering why you chose to be active, with a support message about what to do when feeling overwhelmed and with a support message about staying positive in times of self-doubt and how to manage a lapse in behavior A video of preteen and teenage girls sharing their experiences and how they overcame challenges with being active. A video of a famous female sports star discussing their role models, how they overcame challenges related to staying active, and who supported them along the way. A video demonstrating ways to stay active at home An infographic with a question about tips to help girls be active A digital badge of congratulations for reaching the end of the module	Knowledge Skills Identity Beliefs about capabilities Beliefs about consequences Intentions Social influences Emotion
App module 7: let us get moving	Includes infographics about the module’s objectives, goal setting what is it, why and how to set goals, the challenges associated with goal setting and how to overcome them, and showing girls how they can monitor their progress in the app Provide a selection of goals related to leisure time PA for girls to choose from Girls select a goal of their choice, including when and where the activity would be enacted and with whom, and a digital badge wishing girls “good luck” with their chosen goal	Knowledge Skills Beliefs about capabilities Beliefs about consequences Intentions Goals Behavioral regulation
App module 8: booster module (included after feedback from the co-design workshops, session 2)	Includes infographics about the module’s objectives; revision of PA behaviors; revision of benefits of being active; how to overcome potential barriers to providing support, for example, using if...then statements; and how lapses in behaviors are normal A video with support messages from other teenage girls and a digital badge of congratulations for reaching the end of the module	Knowledge Identity Beliefs about capabilities Beliefs about consequences Intentions Social influences Emotion Behavioral regulation
App module 9: final module	Infographics about the module’s objective; recap on PA and benefits of PA; recap on goal setting; and recap on how to overcome challenges, where to look for social support, and what to do next A survey with questions about PA levels and being active. A survey providing feedback about acceptability and feasibility of the study and a digital badge of congratulations for reaching the end of the module	Knowledge Beliefs about consequences Intentions Behavioral regulation
App icon: resources	Links to websites of resources available to them in their local area Podcasts about PA, with a focus on girls Videos of other preteen girls and their experiences	Knowledge Skills Beliefs about capabilities Beliefs about consequences Intentions Social influences Emotion
App icon: goals	Summary of goals set for the duration of the study Girls can review progress of goals set in the module (eg, walk or wheel to school with mother twice a week and practice skills at home) Girls can manually set and record additional goals of their choice	Intentions Goals Behavioral regulation
App icon: trackers	Option available for girls to self-monitor their progress of goals set that are paired with a smartwatch that is synced with the app (eg, step count and exercise minutes) Girls can self-monitor their daily steps and exercise minutes manually Girls manually self-monitor an activity (eg, practiced skills), rate their enjoyment factor, and record any notes or points of interest	Intentions Goals Behavioral regulation
App feature: motivational messages	Tailored prompts or cues sent as push notifications to girls that are age appropriate and relative to their chosen goals. Generic messages of encouragement, praise, or inspiration that change each day	Beliefs about capabilities Identity Social influences Emotion
Week 8: conclusion of study and group meeting (included after feedback from the co-design workshops, session 2)	Face-to-face group meeting with girls to award girls with a certificate of completion, receive feedback on the intervention, and answer any questions	Social influences

#### Intervention Delivery

**Figure 2:**
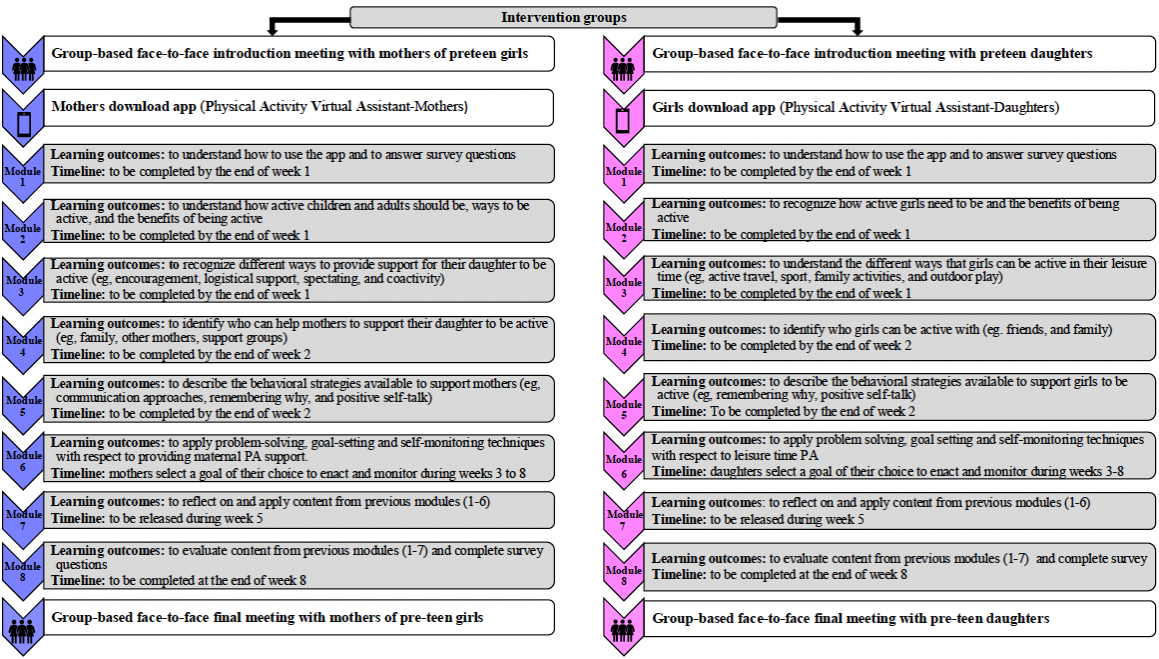
Intervention schedule. PA: physical activity.

Intervention delivery was considered from 4 perspectives: mode of delivery, intervention setting, schedule, and delivery style in line with the BCIO [49]. As described in Phase 2: Identify Intervention Functions, Content, and Implementation Options section, the intervention’s mode of delivery is primarily through a mobile app with a face-to-face component at the start and end of the intervention. The settings where the intervention takes place for mothers and daughters are at their household residences, local sport and exercise facilities, or in outdoor environments (ie, local parks, greens, forests, or beaches). The time frame chosen for the intervention schedule is based on the findings from formative research, which suggested that mother-daughter interventions lasting <12 weeks were likely to be more effective [82], and from engagement with participants in the co-design sessions and the academic advisory panel. The 8-week intervention schedule starts with face-to-face sessions for both mother and daughter participants. Over the course of the first 2 weeks of the intervention, 5 short modules are released for the participants to complete. Following completion of the modules, both mothers and daughters are then required to select and set a goal of their choice related to the target behavior (module 6). They then self-monitor their progress for 6 weeks. A booster module summarizing the intervention content is released during week 5 of the intervention, and there is a final module to be completed at the end of the intervention. To conclude the intervention and answer any questions, a second face-to-face session is held with the mothers and daughters. Figure 2 provides an overview of the intervention schedule and details of the core learning outcomes of the modules for both apps.

To ensure the communication style in which the intervention content (ie, BCTs) is delivered is collaborative, autonomy supportive, and person centered [48], the principles of SDT [85] were applied. According to SDT, autonomous motivation for a behavior is developed through the satisfaction of the basic psychological needs of autonomy, competence, and relatedness [85]. The need for autonomy refers to a mother’s or daughter’s desire to have choice and to feel empowered in directing their own behavior [85]. For example, in the app, the goal-setting feature supports the basic need of autonomy by providing mothers and daughters with choices and options, enabling them to make decisions and take responsibility about how they chose to support their daughter or be active. The need for competence relates to a mother’s or daughter’s need to feel capable of achieving a desired outcome [85]. To illustrate, whenever mothers or daughters log activities on the app, it represents a confirmation that they sustained the behavior and thus enhances their feelings of competence. The need for relatedness denotes an individual’s aspirations to feel a sense of belonging and connectedness with others [85]. For instance, the messaging feature enables mothers to connect with others who face the same challenges or achieve the same goals, thus promoting a sense of belonging and providing an opportunity to develop meaningful relations with other participants (Table 6).

**Table 6:** Intervention delivery style, illustrating how app features align with the principles of self-determination theory.

App features		Description	Expected benefit
**Autonomy-supportive features**			
	Goal setting	This feature provides mothers and daughters with the option to choose from a set of predefined activities or exercises they wish to perform Mothers and daughters have the option to proactively set a goal they will perform, which is related to either maternal support for PAa or daughters’ leisure time activity	The goal-setting feature supports the basic need of autonomy and promotes autonomous motivation by providing mothers and daughters with choices and options, enabling them to make decisions and take responsibility about how they choose to support their daughter and be active
	Reminders	The app will provide a reminder that is delivered as a push notification around the time the mothers and daughters should perform a specific activity The reminders are set by the mothers and daughters while they are selecting their goals and are optional	This feature helps mothers and daughters stay organized and on track with regard to the target behaviors To reduce the feeling of acting out of pressure or control, this is an optional feature and can only be activated by the mothers and daughters
	Motivational messages	Feature with preset messages delivered as push notifications that provide encouragement, praise, and inspiration to perform target behaviors Messages are not task inherent and are provided to mothers and daughters at specified time intervals regardless of performance or completion of target behaviors A feature that allows mothers and daughters to write a brief message about why it is important for them to continue engaging in the target behavior. This self-directed message is available whenever needed and can be delivered as a push notification at chosen time intervals	Mothers and daughters provided feedback regarding the time and type of messages they would like to receive in an earlier module; the messages are tailored to suit their preferences The messages provide a meaningful rationale for engaging in the target behaviors The self-directed messages enable mothers and daughters to reflect on why they want to engage and sustain the behaviors
**Competence-supportive features**			
	Self-monitoring	Provides mothers and daughters with option to self-record the accomplishment of a goal or the completion of a task related to the target behaviors Mothers and daughters can record information about what happened on specific days (eg, bad weather, lots of homework, and stress at work) and rate their enjoyment factor while partaking	Whenever mothers or daughters log an activity, it represents a confirmation that they sustained the behavior and thus enhances their feelings of competence The information entered helps mothers and daughters know themselves and understand their personal circumstances that influence the target behavior By entering data into the app, mothers and daughters express their interest in maintaining the behaviors
	Activity feedback	Provides mothers and daughters with information about how the task that was performed and provides them with details of their overall progress toward completing a predefined set of activities or goal. The information might be accompanied by a score (eg, step count) or encouragement message or badge (well done for completing the module) Timing of feedback is important to avoid unsatisfactory results such as underachievement; therefore, mothers and daughters choose to view their own feedback rather than receiving it unexpectedly The activity feedback needs to be personal, nonevaluative and specific to the task performed.	Positive feedback shows growth or improvement trends and enhance mothers’ and daughters’ sense of competence Activity feedback in the form of encouragement messages or badges can foster positive emotions toward the target behavior
**Relatedness-supportive features**			
	Community forum	Enables mothers and daughters to connect with other participants where they have the opportunity to interact and connect with others The research team will also facilitate opportunities for participants to meet and participate in activities through the group chat feature	Messaging enables mothers and daughters to connect with other participants who face the same challenges They can share experiences, provide and receive support, and experience a sense of belonging and relatedness
	Modeling videos or podcasts	Videos or podcasts of other mothers of preteen daughters and preteen girls sharing their experiences are embedded throughout the app’s modules and in the resources icon	These videos or podcasts provide mothers and girls with information and advice from other mothers and daughters who face similar challenges, which can help satisfy their need for relatedness and support their autonomous motivation to perform the target behavior over time

^a^PA: physical activity.

#### Feedback From Co-Design Workshops and Intervention Refinement

After the development of the intervention content and delivery (with separate mobile apps for mothers and daughters), a second series of workshops was held to present the mobile apps to mothers, preteen girls, and teachers. All groups acknowledged how the intervention content was informative and persuasive, as shared by Sadie, fifth class:

Instead of just getting girls to join sports, giving good reasons as well. Instead of saying like, do you want to try this and try this? It was giving good reasons.

The girls found the videos of other girls’ experiences regarding being active useful and inspiring, particularly those with girls their own age and a little older than them, as described by Sophie, sixth class:

Because if they’re girls older, like what Evie said, they can be like role models. If they’re the same age as you, then they could inspire you to join a team as well.

This was a similar finding for the mothers and teachers, who recognized how videos demonstrating experiences of “other mothers and girls they can relate to” (Kate, primary school teacher) would encourage maternal PA support and girls to be active. The mothers’ and teachers’ groups provided positive feedback when exploring the resources feature, which presented what was available to them in their local community for supporting their daughter to be active, as described by Susan, who has a daughter in sixth class:

That’s brilliant. Little bits like that on it, You just let people know (about the resources feature) and you just click the link then and pick it up.

Several amendments were suggested at these workshops, which were then included in the final version of the intervention. For example, the mothers and teachers’ groups suggested that it would be important to have an initial and final group-based face-to-face session as part of the intervention, as shared by Emer, a primary school teacher:

I think at the start, if you get them in like...that first meeting and first introduction thing is crucial. They feel invested in it.

As a result of this feedback, we introduced both an initial and final group-based face-to-face intervention sessions. Specifically, the initial session will enable mothers and preteen daughters to meet other users of the smartphone app and develop social connections, which can then be reinforced through using some of the social support features on the app. It would also allow mothers and preteen daughters to get instruction from the research team as to how to use the features of the smartphone app. The final face-to-face session will allow mothers and preteen daughters to share their experiences and provide an opportunity to sustain their social network developed as part of the intervention. It was also suggested to avoid providing all the modules on the app at once, instead phasing them in over a few weeks to prevent mothers and daughters from feeling overwhelmed by the information. This recommendation was shared by Michelle, whose daughter was in fourth class:

I’d phase it in, different bits of information every couple of weeks...I think if you throw too much at people, they won’t bother looking at it. It’s just too much information...People don’t like too much information at once. It just bugs them.

Further suggestions were for a booster module to be added to the app to provide a reminder of the key features of the intervention content and for a podcast with parenting tips for teenage daughters to be added to the resources feature as “there’s a lot of challenges out there. People are looking for...Looking for help and guidance.” (Jennifer, daughter in sixth class).

#### Evaluation of the Intervention Prototype and Logic Model

**Figure 3:**
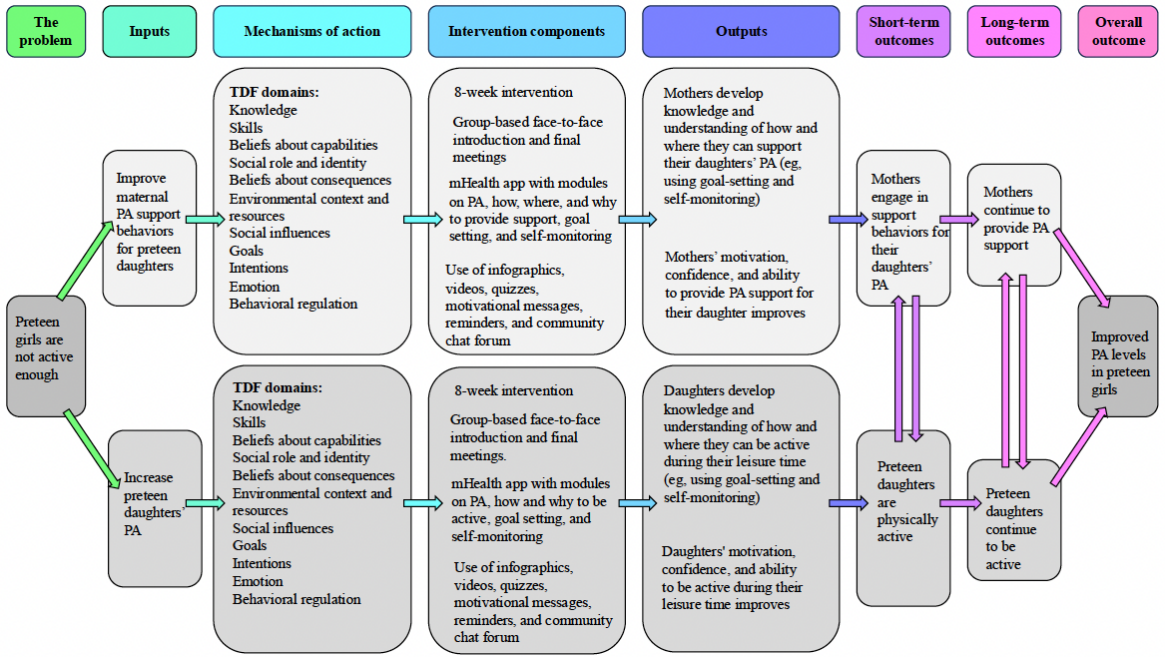
Logic model of the intervention prototype. mHealth: mobile health; PA: physical activity; TDF: Theoretical Domains Framework.

**Figure 4:**
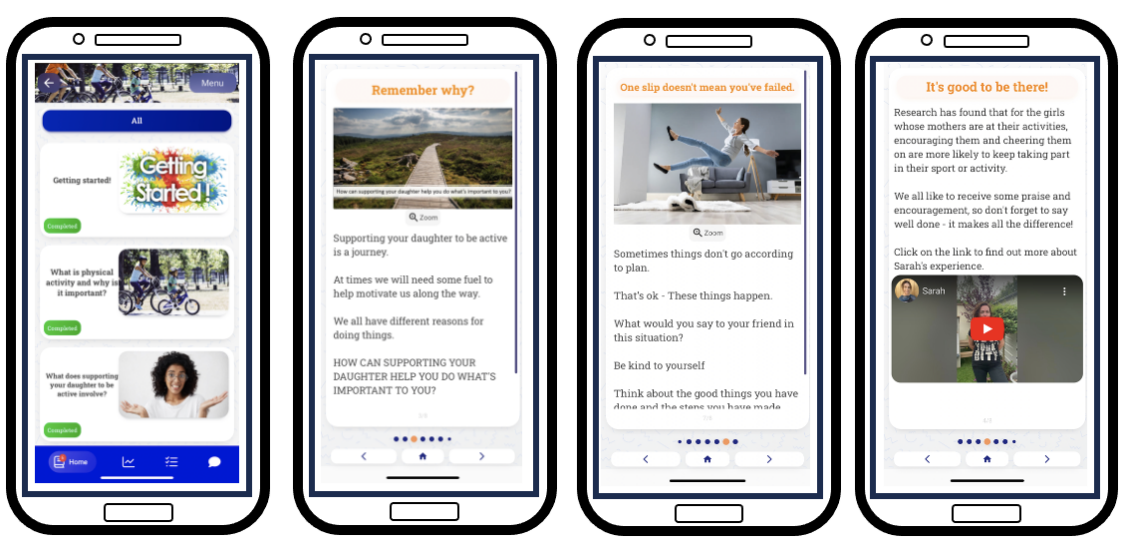
Screenshots from the mothers’ mobile app prototype.

**Figure 5:**
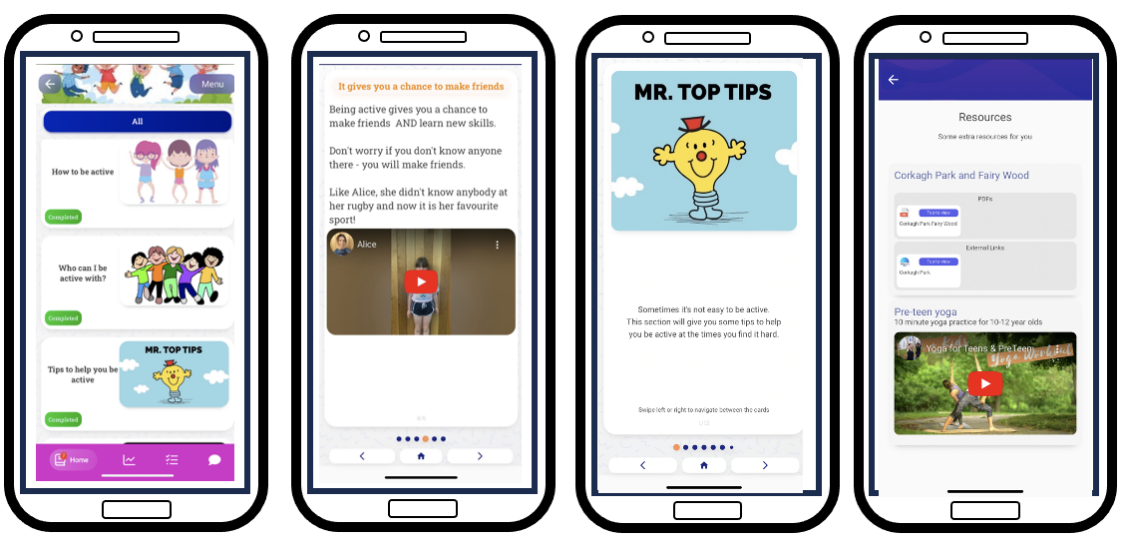
Screenshots from the daughters’ mobile app prototype.

The App Behavior Change Scale [78] was used as a checklist by the research team to assess the behavior change elements of the apps. Both proposed mobile apps included 18 items on the scale, indicating a high number of BCTs embedded in the apps and strong behavior change potential (Multimedia Appendix 5 [78]). The academic advisory panel reviewed and agreed on the final intervention prototype as well as the refined program theory. Figure 3 is a logic model that represents the program theory of the mother-daughter intervention. It depicts the flow of the intervention from (1) the identification of the problem (ie, preteen girls are not active enough) to (2) the inputs (ie, target behaviors of maternal PA support and preteen girls’ PA), to (3) the mechanisms of action (ie, Table 1), to (4) the intervention components (ie, Tables 4 and 5), to (5) outputs (ie, mothers and daughters develop knowledge and understanding and improve motivation to enact target behaviors), to (6) short-term outcomes (ie, mothers and daughters enact target behaviors), to (7) long-term outcomes (ie, mothers and daughters maintain target behaviors), and finally (8) overall outcomes of the intervention (ie, improved PA levels in preteen girls). The app targeting mothers will be called the Physical Activity Virtual Assistant for Mothers (PAVA-M), whereas the app targeting their preteen daughters will be called Physical Activity Virtual Assistant for Daughters (PAVA-D) (Figures 4 and 5).

## Discussion

### Principal Findings

This paper describes the systematic process to develop an evidence- and theory-informed intervention, using co-design methods, to increase PA in preteen girls of low SEP by incorporating maternal supportive behaviors. This is noteworthy given that levels of PA decline with age in preteen girls of low SEP [4,9], placing them at elevated risk of obesity, type 2 diabetes, and cardiovascular disease [8,10]. In keeping with MRC guidance, the intervention was refined through an iterative and dynamic process based on evidence, theory, feedback from co-design workshops with mothers of preteen daughters, preteen girls, and primary school teachers and input from a multidisciplinary academic advisory panel. This process resulted in the development of an intervention with 2 target behaviors, one targeting mothers’ supportive behaviors for their daughters’ PA and the other targeting preteen daughters’ PA directly, which is ready for feasibility and acceptability testing.

The systematic approach applied in this study was guided by the BCW framework for developing interventions [37]. Using the BCW facilitated a rigorous analysis of the problem and how it could be potentially addressed. It also enabled the consideration and incorporation of evidence from several sources: the extant research literature, formative research [82-84], as well as the judgments of the academic advisory panel. We followed a step-by-step process that involved the following: identifying and specifying the target behaviors; conducting a thorough analysis of the barriers and enablers to these behaviors; using the TDF to identify the proposed mechanisms of action; and selecting feasible intervention functions, BCTs, and delivery methods. One study has used the BCW to develop a mother-daughter PA intervention for adolescent girls [41], but to our knowledge, this is the first study to use the BCW and TDF in conjunction with the BCIO to target children’s PA through a theory-informed family-based intervention.

The intervention prototype incorporates 27 BCTs, which is greater than the average of 8 to 10 BCTs per intervention reported in recent systematic reviews of family-based interventions targeting health behaviors such as PA [82,88]. There is some evidence to suggest that more effective interventions include a greater number of BCTs [89]. Furthermore, interventions that include a greater number of BCT clusters, with a threshold of at least 3 clusters, increase the likelihood of intervention effectiveness [90]. There were 13 BCTs clusters within the intervention prototype, and we incorporated particular clusters and specific techniques that have shown promise in theory-based interventions. For example, identity is an important mechanism of action for the promotion and maintenance of PA in adults and young people [63,91] and for providing parental PA support [64]. Our intervention is one of the few to include BCTs, which strengthen maternal identity for PA support and mother and daughter PA identity such as “reframe past behaviour BCT,” “identify self as role model BCT,” and “adopt changed self-identity BCT” [64]. In addition, this study incorporates BCTs that have proven effective in mother-daughter PA interventions and more broadly in health behavior change research. These include selecting a relevant behavioral goal, self-monitoring progress toward that goal, and developing problem-solving skills to address potential challenges [82,92-94].

This study engaged with end users (eg, mothers and preteen girls) and other relevant stakeholders (eg, primary school teachers) in the intervention development process using co-design methods. Despite continued advocacy for engaging children and adolescents in co-design methods, there is a paucity of studies targeting family-based PA that have applied such methods, in particular when it comes to children aged 10 to 12 years [59]. To the best of our knowledge, this is the first intervention prototype that meaningfully engaged with girls aged 10 to 12 years throughout the entire development process. The girls provided information into the selection of intervention components and towards the acceptability of intervention materials and resources [95]. Interestingly, the girls in the study suggested that a video of teenage girls slightly older than they were (ie, aged 13-14 years) describing how they overcame challenges to PA would be relatable and helpful for promoting PA in their cohort, an approach that the research team had not considered. Therefore, by including girls aged 10 to 12 years in the co-design process, this study increased the likelihood of acceptability and implementation at the intervention testing stage [56,96]. Furthermore, there is a lack of resources dedicated to detailing and evaluating the process of engaging with participants using co-design methods in the development of interventions [55,97]. As a result, there may be a need to develop guidance as to how to report the use of co-design principles in studies similar to the Template for Intervention Description and Replication (TIDieR) checklist [98] or the BCIO [46].

The mode of delivery of the intervention was another important intervention component. The selection of the mobile app was driven by the end users who wanted flexibility in how they engaged with the intervention. Indeed, mothers in the study highlighted the importance of being able to complete the intervention at their own pace, thus recommending a mobile app as the primary mode of delivery. Mothers often describe barriers to engaging in PA related to household, family, and occupational responsibilities [83]. Thus, the mobile app may allow individuals to complete intervention content at their own pace and facilitate adherence to the intervention. There is increased use of mobile apps as a mode of delivery for PA interventions [22,99,100]. However, research to date in children and adolescent populations is less frequent and is typically poorly designed [100]. Consequently, there is a need for further systematic theoretically informed research on the use of mobile apps with this population, a need which this study attempts to address. One of the challenges in using mobile apps as the mode of delivery for interventions is the cost of development of such apps, which can be prohibitive [101]. This study used the Pathverse platform to address this issue, as it provided our team with a rapid and cost-effective tool for creating and refining the intervention content [86,102]. Alongside the use of the BCW and related elements, we used the App Behavior Change Scale as a checklist during the development of the intervention to maximize the behavior change potential of the applications. However, it is important to note that the App Behavior Change Scale only measures the theoretical behavior change potential of the application, and it does not attempt to investigate the relationship between the actual features of application and behavioral outcomes [39]. Future work should consider the uptake, engagement, and user retention of the app by following frameworks such as the Reach, Effectiveness, Adoption, Implementation, and Maintenance framework [103].

An important component within intervention development is how an intervention is delivered, including the style of delivery of the intervention [48]. Typically, this focuses on human to human interaction; however, there is an increasing realization of the importance of considering a person-centered intervention delivery style, which is reflective and empathetic when designing applications and their related content [104]. Consequently, the principles of SDT [85] were applied to the intervention, ensuring that BCTs and specific features used in the intervention mapped to the basic psychological needs of autonomy, competence, and relatedness proposed by SDT [105-107]. Indeed, there is a growing body of work highlighting how applications underpinned by the SDT principles can strengthen digital therapeutic alliance and increase engagement in behaviors such as PA [107,108].

### Future Directions

This study took place within the intervention development phase of the MRC framework [32]. Future research would involve using a no-code development app [102] to assess the feasibility of the intervention and inform decisions about how to progress to the following phases of intervention evaluation and implementation [32]. After engagement with the co-design participants, it was suggested that it was most feasible to promote this intervention via the school environment although it is targeting girls’ PA outside of school hours. The school setting can reach children and adolescents of diverse racial and socioeconomic backgrounds and provides a ready-made social network for both mothers and daughters to engage with when undertaking the intervention [109,110]. Furthermore, it would allow for tailoring of the intervention resources within the school and local community that could support increased leisure time PA [109,110]. This is in line with research by Pfledderer et al [111] and van Sluijs et al [23] who recommend that interventions consider both family and community engagement (eg, family based and linked to school) to promote children’s and adolescents’ PA, particularly for underserved populations such as children and adolescents of low SEP. Although our preference is for mothers and daughters to take part in the intervention, the separate mobile app mode of delivery allows preteen girls to partake in the intervention regardless of their mothers’ participation. This is an important feature, given that reaching parents of low SEP is often a challenge for interventions [112].

A limited number of these interventions are scaled-up and applied in real-world settings, identifying a significant research practice gap [113,114]. A recent review by Crane et al [115] found that health interventions (including PA interventions) that followed a research pathway were approximately 3 times more likely to have a positive effect on population health. Therefore, in line with recommendations by McKay et al [113], our future research would involve continuous planning for scaling-up, developing scale-up pathways, and evaluation of the scale-up throughout the duration of the intervention. Schools serving children with low SEP are frequently underresourced and often need more support to reach the same outcomes as their more advantaged counterparts [109,113]. To this end, maintaining relationships with schools and local community partners is essential in the scaling-up process to establish trust and identify potential implementation barriers [113,114]. This would involve hosting meetings with principals, teachers, and administrators to understand the pressing issues in their school environment; engaging with teachers, coaches, and local community partners to overcome implementation barriers; and developing collaborative strategies to encourage mothers and daughters to be physically active and sustain activity levels after the intervention [116]. Finally, based on the findings from this study, potential avenues for future research could be additional studies to evaluate the long-term effectiveness and sustainability of the intervention, research exploring the factors influencing parental engagement in family-based mHealth interventions, and investigation into the impact of mobile app on PA behavior change in children and adolescents.

### Strengths and Limitations

This study used a systematic, evidence- and theory-based approach to integrate a body of evidence from a systematic review, 2 qualitative studies, an academic advisory panel, and end users’ knowledge to co-design and develop a novel intervention to promote PA in preteen girls of low SEP. The uniqueness of this study lies in following the first phase of the MRC framework, while using the BCW, the TDF, BCTO, and input from co-design workshops, which offered procedural direction, structure, and transparency. Annotating the BCIO entities enabled us to represent the intervention characteristics in a detailed and structured way, which can be used across contexts and disciplines. In addition, the entities’ unique identifiers will facilitate the use of artificial intelligence including machine learning–based methods in data extraction and evidence synthesis [46]. Family-based PA interventions have been the focus of previous research [21,117]. However, no digital intervention to date has specifically focused in promoting PA in preteen girls of low SEP complemented by maternal support behaviors. Thus, this work fills an important gap by seeking to support an at-risk group. Furthermore, the involvement of key stakeholders in the development process is a key strength of this study. It ensures that the content of the intervention was adapted to accommodate the users’ needs, making it useful and relevant, thus increasing the likelihood of a more feasible, acceptable, and ultimately effective intervention [56,118].

Our work has some limitations. First, the highly structured and systematic approach used to develop this intervention prototype takes a significant amount of time and resources. For example, using the BCW, the TDF, and the BCTO requires considerable skills and training. Second, the process of converting BCTs into intervention content can be open to interpretation, and the research team had to make subjective and pragmatic decisions regarding intervention content throughout the process [119,120]. Third, we did not collect additional information regarding mothers’ backgrounds such as their educational levels and PA experience as part of the co-design workshops. Finally, similar to other research [45], we used DEIS schools to recruit low-SEP preteen girls and their mothers. However, the data might not be fully representative of the target population, as DEIS schools are categorized by district, and it is possible that some girls or mothers in the school might not be of low SEP. Continued efforts should be made to target this cohort, for example, using household income or area level socioeconomic status.

### Conclusions

In conclusion, this study uses a systematic evidence- and theory-based approach incorporating findings from a systematic review, formative qualitative research with mothers and preteen girls, input from an academic advisory panel, and knowledge from end users. This process was used to co-design an mHealth intervention prototype aimed at promoting PA in preteen girls, with a focus on maternal support behaviors, and is now ready for feasibility and acceptability testing. The novel contribution of this study lies in the use of theory and the meaningful involvement of key stakeholders throughout the development process. In addition, this study offers a practical example of how to integrate evidence, theory, and stakeholder engagement, which can be adjusted and tailored to fit different contexts and populations. Finally, the comprehensive annotation of the BCIO entities denotes the intervention characteristics in a structured manner that enables improved communication, replication, and implementation of interventions.

## Appendix

Multimedia Appendix 1 Behavior change intervention glossary.

Multimedia Appendix 2 Barriers and enablers to target behaviors.

Multimedia Appendix 3 Co-design session 1.

Multimedia Appendix 4 Co-design session 2.

Multimedia Appendix 5 App Behavior Change Scale.

## Abbreviations

BCIO: Behaviour Change Intervention Ontology
BCT: behavior change technique
BCTO: Behaviour Change Technique Ontology
BCW: Behaviour Change Wheel
COM-B: Capability, Opportunity, and Motivation–Behavior
mHealth: mobile health
MRC: Medical Research Council
PA: physical activity
PAVA-D: Physical Activity Virtual Assistant for Daughters
PAVA-M: Physical Activity Virtual Assistant for Mothers
SDT: self-determination theory
SEP: socioeconomic position
TDF: Theoretical Domains Framework
TIDieR: Template for Intervention Description and Replication
