# Supplementary material for: Developing an Evidence- and Theory-Informed Mother-Daughter mHealth Intervention Prototype Targeting Physical Activity in Preteen Girls of Low Socioeconomic Position: Multiphase Co-Design Study
Source: JMIR Pediatr Parent. 2025 Jan 6;8:e62795. doi: 10.2196/62795 (PMC11747544; doi:10.2196/62795)
Supplement: Multimedia Appendix 1 [file pediatrics_v8i1e62795_app1.docx]

Table 1 Behaviour Change Intervention Entities used in mother-daughter PA intervention

| **Label** | **Unique Identifier** | **Definition** |
| --- | --- | --- |
| autonomy-supportive  communication style | [[BCIO:044084]](https://bciosearch.org/BCIO_044084) | A communication style which aims to promote the recipient’s capacity to make their own decisions. |
| behaviour change intervention | [[BCIO:003000]](https://bciosearch.org/BCIO_003000) | An intervention that has the aim of influencing human behaviour. |
| behaviour change intervention content | [[BCIO:007000]](https://bciosearch.org/BCIO_007000) | An intervention content that is part of a behaviour change intervention. |
| behaviour change intervention delivery | [[BCIO:008000]](https://bciosearch.org/BCIO_008000) | An intervention delivery in which the intervention is a behaviour change intervention. |
| behaviour change intervention mechanism of action | [[BCIO:006000]](https://bciosearch.org/BCIO_006000) | A process that is causally active in the relationship between a BCI scenario and its outcome behaviour. |
| behaviour change intervention mode of delivery | [[BCIO:011000]](https://bciosearch.org/BCIO_011000) | An attribute of a BCI delivery that is the physical or informational medium through which a BCI is provided. |
| behaviour change intervention schedule of delivery | [[BCIO:009000]](https://bciosearch.org/BCIO_009000) | A BCI attribute that involves its temporal organisation. |
| behaviour change intervention style of delivery | [[BCIO:044000]](https://bciosearch.org/BCIO_044000) | A communication style that is an attribute of a BCI content communication process |
| behaviour change technique (BCT) | [[BCIO:033000]](https://bciosearch.org/BCIO_033000) | A planned process that is the smallest part of BCI content that is observable, replicable and on its own has the potential to bring about behaviour change. |
| collaborative  communication style | [[BCIO:044085]](https://bciosearch.org/BCIO_044085) | A communication style characterised by a sense of the initiator and recipient working together, the initiator showing sensitivity to the others’ needs, in order to obtain an outcome desired by all. |
| communication style | [[BCIO:044004]](https://bciosearch.org/BCIO_044004) | A particular manner of communicating aimed at inducing or avoiding certain kinds of responses in others, or demonstrating certain characteristics of the initiator. |
| Face-to-face mode of  delivery | [[BCIO:011003]](https://bciosearch.org/BCIO_011003) | Human interactional mode of delivery that involves an intervention source and recipient being together in the same location and communicating directly. |
| Group-based mode of  Delivery | [[BCIO:011057]](https://bciosearch.org/BCIO_011057) | Mode of delivery that involves three or more people in the location where the intervention is delivered. |
| Household residence | [[BCIO:026009]](https://bciosearch.org/BCIO_026009) | A facility where an individual is living alone or with one or more person. The individuals do not have to be related. |
| Intervention style of  delivery | [[BCIO:044005]](https://bciosearch.org/BCIO_044005) | A communication style that is an attribute of an intervention content communication process. |
| Mobile application mode of delivery | [[BCIO:011028]](https://bciosearch.org/BCIO_011028) | Electronic mode of delivery that involves the intervention recipient interacting with a mobile application. |
| Outdoor environment | [[BCIO:026044]](https://bciosearch.org/BCIO_026044) | A site which is an outdoor location outside of a building. |
| person-centred  intervention delivery | [[BCIO:044006]](https://bciosearch.org/BCIO_044006) | An intervention delivery characterised by efforts by an intervention source to give a person the resources they need to manage their own life and make them an active participant in deciding how to move forward. |
| Push mode of delivery | [[BCIO:011062]](https://bciosearch.org/BCIO_011062) | Mode of delivery that is not dependent  on actions on the part of the intervention recipient. |
| Sport and exercise facility | [[BCIO:026030]](https://bciosearch.org/BCIO_026030) | A community facility used for exercising. Example: gym, stadium, tennis courts, swimming pool |
| Synchronous mode of  delivery | [[BCIO:011060]](https://bciosearch.org/BCIO_011060) | Mode of delivery that involves delivery and receipt of the intervention or its components occurring at the same time  or very close in time. |

Table 2 BCTs used in mother-daughter PA intervention

| **BCT Group and ID** | **BCT Group Definition** | **BCT Label and ID** | **BCT Definition** |
| --- | --- | --- | --- |
| Goal directed BCT  [[BCIO:007001]](https://bciosearch.org/BCIO_007001) | A <behaviour change technique> that sets or changes goals. | Action planning BCT [[BCIO:007010]](https://bciosearch.org/BCIO_007010) | A <goal directed BCT> that involves making a detailed plan for the performance of the behaviour, which must include at least one of context, frequency, duration or intensity. |
|  |  | Goal strategising BCT  [[BCIO:007008]](https://bciosearch.org/BCIO_007008) | A <goal directed BCT> in which the person analyses factors influencing the behaviour and generates, selects, or reviews strategies to increase facilitators and overcome barriers. |
|  |  | Set behaviour goal BCT  [[BCIO:007003]](https://bciosearch.org/BCIO_007003) | A goal setting BCT that sets a goal for the behaviour to be achieved. |
|  |  | Set graded tasks BCT  [[BCIO:007100]](https://bciosearch.org/BCIO_007100) | A <goal directed BCT> that sets easy-to-perform tasks for the person, making them increasingly difficult, but achievable, until the behaviour is performed |
| Monitoring BCT [[BCIO:007017]](https://bciosearch.org/BCIO_007017) | A <behaviour change technique> that involves gathering or using information about performance. | Monitor emotional consequences BCT  [[BCIO:007066]](https://bciosearch.org/BCIO_007066) | A <monitoring BCT> that involves the person assessing their emotions after performing the behaviour. |
|  |  | Provide feedback on behaviour BCT  [[BCIO:007023]](https://bciosearch.org/BCIO_007023) | A <provide feedback BCT> that provides information about the person's previous performance of the behaviour. |
|  |  | Self-monitor behaviour BCT  [[BCIO:007024]](https://bciosearch.org/BCIO_007024) | A monitoring BCT in which the person uses a method to monitor and record their behaviour. |
| Social support BCT  [[BCIO:007028]](https://bciosearch.org/BCIO_007028) | A behaviour change technique that involves taking steps to secure or deliver the support or aid of another person. |  |  |
| Guide how to perform behaviour BCT [[BCIO:007050]](https://bciosearch.org/BCIO_007050) | A <behaviour change technique> that provides guidance regarding how to perform the behaviour. | Demonstrate the behaviour BCT  [[BCIO:007055]](https://bciosearch.org/BCIO_007055) | A <guide how to perform behaviour BCT> that provides an observable sample of the performance of the behaviour for the person to aspire to or imitate. |
|  |  | Instruct how to perform behaviour BCT  [[BCIO:007058]](https://bciosearch.org/BCIO_007058) | A <guide how to perform behaviour BCT> that involves telling the person how to perform the behaviour. |
| Suggest different perspective on behaviour BCT [[BCIO:007302]](https://bciosearch.org/BCIO_007302) | A <behaviour change technique> that suggests the deliberate adoption of a new perspective on the behaviour. | Reframe past behaviour BCT  [[BCIO:007056]](https://bciosearch.org/BCIO_007056) | A <suggest different perspective on behaviour BCT> that involves reattributing a person's successes to internal, stable or global factors or failures to external, unstable or specific factors. |
| Increase awareness of consequences BCT [[BCIO:007062]](https://bciosearch.org/BCIO_007062) | A <behaviour change technique> that draws attention to consequences of the behaviour in the normal course of events. | Inform about environmental consequences BCT  [[BCIO:007176]](https://bciosearch.org/BCIO_007176) | An <increase awareness of consequences BCT> that provides information about the environmental consequences of performing or not performing the behaviour. |
|  |  | Inform about health consequences BCT  [[BCIO:007063]](https://bciosearch.org/BCIO_007063) | An <increase awareness of consequences BCT> that provides information about the physical or mental health consequences of performing or not performing the behaviour. |
|  |  | Inform about social consequences BCT  [[BCIO:007064]](https://bciosearch.org/BCIO_007064) | An <increase awareness of consequences BCT> that provides information about the social consequences of performing or not performing the behaviour. |
| Awareness of other people’s thoughts, feelings and actions BCT [[BCIO:007072]](https://bciosearch.org/BCIO_007072) | A <behaviour change technique> that increases awareness of what other people think, do, or feel. | Present information from credible influence BCT  [[BCIO:007075]](https://bciosearch.org/BCIO_007075) | An <awareness of other people's thoughts, feelings and actions BCT> that presents information from a credible person or organisation to influence the behaviour. |
|  |  | Prompt social comparison BCT  [[BCIO:007073]](https://bciosearch.org/BCIO_007073) | An <awareness of other people's thoughts, feelings and actions BCT> that draws attention to other people's behaviour and compares it with the person's own behaviour. |
| Advise specific behaviour BCT [[BCIO:007168]](https://bciosearch.org/BCIO_007168) | A <behaviour change technique> that advises the person to perform a behaviour in a particular way to help change the target behaviour. | Context-specific repetition of behaviour BCT  [[BCIO:007096]](https://bciosearch.org/BCIO_007096) | An <advise specific behaviour> BCT that advises the person to repeat the behaviour in the same context. |
|  |  | Practise behaviour BCT  [[BCIO:007094]](https://bciosearch.org/BCIO_007094) | An <advise specific behaviour BCT> that advises repetition of the behaviour in a way that has the function of increasing the skill in performing the behaviour. |
|  |  | Substitute behaviour BCT  [[BCIO:007095]](https://bciosearch.org/BCIO_007095) | An <advise specific behaviour BCT> that advises the person to replace the unwanted behaviour with another behaviour. |
| Prompt thinking related to successful performance BCT [[BCIO:007239]](https://bciosearch.org/BCIO_007239) | A <behaviour change technique> that prompts thinking relating to successful performance of the behaviour. | Persuade about personal capability  [[BCIO:007137]](https://bciosearch.org/BCIO_007137) | A <prompt thinking related to successful performance BCT> that persuades the person that they can successfully perform the behaviour. |
|  |  | Prompt focus on past success BCT  [[BCIO:007139]](https://bciosearch.org/BCIO_007139) | A <prompt thinking related to successful performance BCT> that prompts the person to think about previous successful performance of the behaviour. |
|  |  | Prompt self-talk BCT  [[BCIO:007140]](https://bciosearch.org/BCIO_007140) | A <prompt thinking related to successful performance BCT> that promotes the use of positive self-talk before or during the behaviour. |
| Advise how to change emotions BCT [[BCIO:007147]](https://bciosearch.org/BCIO_007147) | A <behaviour change technique> that suggests a method to alter emotions. | Advise how to reduce negative emotions BCT  [[BCIO:050344]](https://bciosearch.org/BCIO_050344) | An <advise how to change emotions BCT> suggesting a method to decrease negative emotions. |
| Restructure the physical environment BCT[[BCIO:050348]](https://bciosearch.org/BCIO_050348) | A <restructure the environment BCT> that alters the physical environment in which the behaviour is, or would have been, performed in a way that facilitates or impedes the behaviour | Add objects to the environment BCT [[BCIO:007156]](https://bciosearch.org/BCIO_007156) | A <restructure the physical environment BCT> that adds objects to the person's physical surroundings. |
| Prompt focus on self-identity BCT  [[BCIO:007157]](https://bciosearch.org/BCIO_007157) | A <behaviour change technique> that prompts the person to focus on their mental representation of themself. | Adopt changed self-identity BCT  [[BCIO:007160]](https://bciosearch.org/BCIO_007160) | A <prompt focus on self-identity BCT> that promotes the adoption of a self-identity as someone who engages in the behaviour that is different from their previous behaviour. |
|  |  | Identify self as role model BCT  [[BCIO:007158]](https://bciosearch.org/BCIO_007158) | A <prompt focus on self-identity BCT> that informs the person that their behaviour may be an example to others. |
| Behavioural consequence BCT [[BCIO:007101]](https://bciosearch.org/BCIO_007101) | A <behaviour change technique> that alters the consequences or promised consequences for the behaviour. | Provide positive social consequence for behaviour BCT  [[BCIO:007265]](https://bciosearch.org/BCIO_007265) | A <provide positive consequence for behaviour BCT> where the consequence is an interpersonal process or a proxy interpersonal process. |
