# Supplementary material for: Developing an Evidence- and Theory-Informed Mother-Daughter mHealth Intervention Prototype Targeting Physical Activity in Preteen Girls of Low Socioeconomic Position: Multiphase Co-Design Study
Source: JMIR Pediatr Parent. 2025 Jan 6;8:e62795. doi: 10.2196/62795 (PMC11747544; doi:10.2196/62795)
Supplement: Multimedia Appendix 2 [file pediatrics_v8i1e62795_app2.docx]

Table 1 Mothers' PA support behaviours (e.g. encouragement, logistical support, co-activity, environmental and regulatory support)

| **TDF Domains**  **(Cane et al., 2012)** | **Related theoretical constructs (Cane et al., 2012)** | **Barriers/ enablers to target behaviour** | **Supporting evidence** |
| --- | --- | --- | --- |
| **Knowledge**  (An awareness of the existence of something) | Knowledge (including knowledge of condition /scientific rationale).  Procedural knowledge.  Knowledge of task environment | - Knowledge/lack of knowledge of forms of PA outside of sport. - Knowledge/lack of knowledge of benefits of PA - Knowledge/lack of knowledge of benefits of PA parental support. - Knowledge/ lack of knowledge of how to access information or opportunities to support their daughter or be active themselves. - Knowledge/or lack of knowledge of daughter’s interests. | (Ingram, McCormick, & Gibson, 2021)  (Sohun, MacPhail, & MacDonncha, 2020)  (Shannon, 2014)  (Rhodes, Perdew, & Malli, 2020)  (Rhodes & Lim, 2018)  Study 2 of PhD |
| **Skills**  (An ability or proficiency acquired through practice | Skills  Skills development  Competence Ability Interpersonal skills  Practice  Skill assessment | - Mother’s ability, skill and competence to engage in PA on her own or with her daughter. - Mothers’ health issues can interfere with their ability to be active themselves or with their daughters/ family members (e.g., knee, hip and back problems, heart problems, diabetes) | (Shannon, 2014) |
| **Social/Professional Role and Identity**  (A coherent set of behaviours and displayed personal qualities of an individual in a social or work setting) | Professional identity Professional role  Social identity  Identity  Professional boundaries Professional confidence  Group identity  Leadership  Organisational commitment | - Mothers identity, ‘ethic of care’, role to support their daughters PA/ good motherhood. - Mothers PA identity (I’m sporty/ not sporty) - Mothers identity around PA support behaviours ( I am a ‘sports’ mother/parent) - Mothers perception of daughters’ PA identity (sporty child/ not sporty) | (Ingram, McCormick, & Gibson, 2021)  (McGannon & Schinke, 2013)  (Rhodes et al., 2019)  (Stirrup, Duncombe, & Sandford, 2015)  (Sutcliffe et al., 2021)  Study 2 of PhD |
| **Beliefs about Capabilities**  (Acceptance of the truth, reality, or validity about an ability, talent, or facility that a person can put to constructive use) " | Self-confidence  Perceived competence  Self-efficacy  Perceived behavioural control  Beliefs  Self-esteem  Empowerment  Professional confidence | - Mother’s self-confidence to be active herself. - Mother’s self- confidence to be active with daughter/ family members - Mother’s self-confidence to provide support for daughter’s/ child’s PA e.g., volunteering, coaching, encouragement, spectating. - Mother’s perceived behavioural control (e.g., control over time commitments and responsibilities) | (Ingram, McCormick, & Gibson, 2021)  (McGannon & Schinke, 2013)  (Rhodes, Perdew, & Malli, 2020)  (Rhodes et al., 2016)  Study 2 of PhD |
| **Optimism**  (The confidence that things will happen for the best or that desired goals will be attained) " | Optimism  Pessimism  Unrealistic optimism  Identity | - Mothers' optimism/ pessimism regarding engaging with their daughters to be active together. |  |
| **Beliefs about Consequences**  (Acceptance of the truth, reality, or validity about outcomes of a behaviour in a given situation) " | Beliefs  Outcome expectancies  Characteristics of outcome expectancies  Anticipated regret  Consequents | - Mother’s beliefs that providing support/ co-activity will improve mother-daughter bonding time (e.g., closeness, stronger relationship, good communication between mother-daughter/ parent- child. - Mother’s beliefs that providing support will promote lifelong healthy behaviours/habits for their daughter. (physical and mental health benefits) - Mother’s beliefs that being active herself will model healthy behaviours for their daughter (e.g., lead by example) | (McGannon & Schinke, 2013)  (Rhodes & Lim, 2018)  Study 2 of PhD |
| **Reinforcement**  (Increasing the probability of a response by arranging a dependent relationship, or contingency, between the response and a given stimulus) | Rewards  Incentives  Punishment  Consequents  Reinforcement  Contingencies  Sanctions |  |  |
| **Intentions**  (A conscious decision to  perform a behaviour or a resolve to act in a certain way) | Stability of intentions  Stages of change model  Transtheoretical model and stages of change | - Stability and commitment of intentions to support. | (Rhodes, Perdew, & Malli, 2020)  (Rhodes et al., 2019) |
| **Goals**  (Mental representations of outcomes or end states that an individual wants to achieve) | Goals (distal / proximal)  Goal priority  Goal / target setting  Goals (autonomous / controlled)  Action planning  Implementation intention | - Preparatory planning for PA support - Alignment with other goals | (Rhodes, Perdew, & Malli, 2020)  (Rhodes et al., 2016) |
| **Memory, Attention and Decision Processes**  (The ability to retain information, focus selectively on aspects  of the environment and choose between two or more alternatives) " | Memory  Attention  Attention control  Decision making  Cognitive overload / tiredness |  |  |
| **Environmental Context and Resources**  (Any circumstance of a person's situation or environment that discourages or encourages the development of skills and abilities, independence, social competence, and adaptive behaviour) | Environmental stressors  Resources / material resources  Organisational culture /climate  Salient events / critical incidents  Person x environment interaction  Barriers and facilitators | - The availability (or lack of) of community resources, e.g. extra-curricular sport available through the school, local sports clubs, and community resources such as parks, playgrounds, and swimming pools. - Parental perceptions of safety in the physical environment, e.g. traffic, roads, ‘stranger danger’ and bullying, influences parental decisions regarding their child’s freedom to be active unsupervised near the family home. - Financial constraints (e.g., cost of activity, transportation) - Time constraints (e.g. work and other children’s activities) | (Rhodes, Perdew, & Malli, 2020)  (Rhodes & Lim, 2018)  (Sohun, MacPhail, & MacDonncha, 2020)  (Shannon, 2014)  (Sutcliffe et al., 2021)  Study 2 of PhD |
| **Social influences**  (Those interpersonal processes that can cause individuals to change their thoughts, feelings, or behaviours) | Social pressure  Social norms  Group conformity  Social comparisons  Group norms  Social support  Power  Intergroup conflict  Alienation  Group identity  Modelling | - Support in facilitating PA from partners and wider family members. - Divergent family interests - Relationship between mother and daughter. - Social norms: Providing opportunities for their daughter through PA (social capital). - Mother- peer relationships (e.g., opportunity to engage with other mothers) - The influence (positive/negative) of daughters peers (friends, boys, siblings) | (McGannon & Schinke, 2013)  (Sohun, MacPhail, & MacDonncha, 2020)  (Stirrup, Duncombe, & Sandford, 2015)  (Sutcliffe et al., 2021)  Study 2 of PhD |
| **Emotion**  (A complex reaction pattern, involving experiential, behavioural,  and physiological elements, by which the individual attempts to deal with a personally significant matter or event) | Fear Anxiety Affect Stress Depression Positive / negative affect Burn-out | - Mothers’ enjoyment of (or lack of) PA can affect mothers in their role as facilitators of daughter’s PA or being active with their daughters. - Mothers’ sense of guilt and inadequacy regarding finances related to PA/ sport - Mother’s positive/negative emotions in response to sports related events or co-activity (e.g., pride, excitement, enjoyment, satisfaction frustration) - Mothers’ affective attitude about providing PA support for their daughter. | (Rhodes, Perdew, & Malli, 2020)  (Sohun, MacPhail, & MacDonncha, 2020)  (Shannon, 2014)  (Stirrup, Duncombe, & Sandford, 2015)  (Sutcliffe et al., 2021)  Study 2 of PhD |
| **Behavioural Regulation**  (Anything aimed at managing or changing objectively observed or measured actions) | Self-monitoring  Breaking habit  Action planning. | - Behavioural regulation skills (monitoring, mobilising support, rewards, action planning, preparatory planning, coping planning) - Action planning - Coping planning | (Rhodes, Perdew, & Malli, 2020)  (Rhodes et al., 2016)  Study 2 of PhD |

Table 2 Pre-teen girls leisure time PA (outside of school hours, includes active travel, sports, family activities (e.g. bike rides, walks) and outdoor play)

| **TDF Domain.**  **(Cane et al., 2012)** | **Related theoretical constructs (Cane et al., 2012)** | **Barriers/ enablers to target behaviour** | **Supporting evidence** |
| --- | --- | --- | --- |
| **Knowledge**  (An awareness of the existence of something) | Knowledge (including knowledge of condition /scientific rationale). Procedural knowledge  Knowledge of task environment | - Girls’ knowledge/ lack of knowledge of benefits of PA. - Girls’ knowledge/ lack of knowledge of forms of PA outside of sport (e.g., active travel) | (Duffey et al., 2021)  (Emm-Collison et al., 2022)  Study 3 of PhD |
| **Skills**  (An ability or proficiency acquired through practice | Skills  Skills development  Competence Ability  Interpersonal skills  Practice  Skill assessment | - Girls’ physical ability to perform skills required to be active. | (Duffey et al., 2021)  (Knowles, Niven, & Fawkner, 2011)  Study 3 of PhD |
| **Social/Professional Role and Identity**  (A coherent set of behaviours and displayed personal qualities of an individual in a social or work setting) | Professional identity  Professional role  Social identity  Identity  Professional boundaries Professional confidence  Group identity  Leadership  Organisational commitment | - Girls’ PA identity (I am sporty/not sporty) - Girls’ social identity (girls are not meant to be sporty/ I am from a sporty family) | (Duffey et al., 2021)  (Knowles, Niven, & Fawkner, 2011)  (Rhodes, Kaushal, & Quinlan, 2016)  Study 3 of PhD |
| **Beliefs about Capabilities**  (Acceptance of the truth, reality, or validity about an ability, talent, or facility that a person can put to constructive use) " | Self-confidence  Perceived competence  Self-efficacy  Perceived behavioural control  Beliefs  Self-esteem  Empowerment  Professional confidence | - Girls’ self-confidence to be active on their own or with others (e.g., friends, family members, boys) | (Cowley et al., 2021)  (Duffey et al., 2021)  (Emm-Collison et al., 2022)  (Knowles, Niven, & Fawkner, 2011)  (Rhodes, Cox, & Sayar, 2022)  Study 3 of PhD |
| **Optimism**  (The confidence that things will happen for the best or that desired goals will be attained) " | Self-confidence  Perceived competence  Self-efficacy  Perceived behavioural control  Beliefs  Self-esteem  Empowerment  Professional confidence | - Girls pessimism around being active with boys (e.g. they won't pass the ball) |  |
| **Beliefs about Consequences**  (Acceptance of the truth, reality, or validity about outcomes of a behaviour in a given situation) " | Beliefs  Outcome expectancies  Characteristics of outcome expectancies  Anticipated regret  Consequents | - Girls' expectations/ beliefs that PA would (not) be enjoyable/fun. | (Duffey et al., 2021)  (Rhodes, Cox, & Sayar, 2022)  Study 3 of PhD |
| **Reinforcement**  (Increasing the probability of a response by arranging a dependent relationship, or contingency, between the response and a given stimulus) " | Rewards  Incentives  Punishment  Consequents  Reinforcement  Contingencies  Sanctions |  |  |
| **Intentions**  (A conscious decision to perform a behaviour or a resolve to act in a certain way) | Stability of intentions  Stages of change model  Transtheoretical model and stages of change |  |  |
| **Goals**  (Mental representations of outcomes or end states that an individual wants to achieve) | Goals (distal / proximal)  Goal priority  Goal / target setting  Goals (autonomous / controlled)  Action planning  Implementation intention |  |  |
| **Memory, Attention and Decision Processes**  (The ability to retain information, focus selectively on aspects  of the environment and choose between two or more alternatives) | Memory  Attention  Attention control  Decision making  Cognitive overload / tiredness | - Girls’ having choice over the activities they undertake. | (Emm-Collison et al., 2022) |
| **Environmental Context and Resources**  (Any circumstance of a person's situation or environment that discourages or encourages the development of skills and  abilities, independence, social competence, and adaptive behaviour) " | Environmental stressors  Resources / material resources  Organisational culture /climate  Salient events / critical incidents  Person x environment interaction  Barriers and facilitators" | - Limited access for girls to sports/ PA programs in the community (especially rural areas) - Opportunities for girls to be active including active travel to/from school, unstructured play and extra-curricular activities. - Competing priorities e.g., homework, socialising with friends. - School holidays (girls less active during holidays) - Weather conditions (can be a barrier or facilitator) - Time constraints - Cost - Competition (girls enjoy being competitive/girls don’t like competing) - Gender bias in sport/ perceived lack of opportunity for females in sport | (Cowley et al., 2021)  (Duffey et al., 2021)  (Emm-Collison et al., 2022)  (Knowles, Niven, & Fawkner, 2011)  Study 3 of PhD |
| **Social influences**  (Those interpersonal  processes that can cause individuals to change their thoughts, feelings, or behaviours) " | Social pressure  Social norms  Group conformity  Social comparisons  Group norms  Social support  Power  Intergroup conflict  Alienation  Group identity  Modelling | - Social support from friends, parents, wider family members, teachers, coaches. - Exclusion by boys/ presence of boys - Friends are not active/ dropped out - Friends are active/ take part - Team dynamics in organised sport (friends are on the team/ nobody will pass me the ball) - Parents safety perceptions (not allowed play outside in local area) - Coach/ parent/ teacher behaviour and communication styles - Social comparison, girls comparing themselves negatively to others (e.g., don’t look as good as others, not as good as others) - Social norms, meeting societal expectations (girls are not meant to be sporty) | (Cowley et al., 2021)  (Duffey et al., 2021)  (Emm-Collison et al., 2022)  (Knowles, Niven, & Fawkner, 2011)  Study 3 of PhD |
| **Emotion**  (A complex reaction pattern, involving experiential, behavioural,  and physiological elements, by which the individual attempts to deal with a personally significant matter or event) | Fear Anxiety Affect Stress Depression Positive / negative affect Burn-out | - Girls emotions towards being active themselves (e.g., fun, enjoyment, fear, embarrassment) - Girls emotions towards being active with friends, family members or boys (e.g., fun, enjoyment, fear, embarrassment) - Girls emotions associated with receiving PA support from parents, friends, teachers, or coaches (e.g., pride, enjoyment, embarrassment, frustration) - Positive/ negative feelings associated with being active with friends, family members. | (Cowley et al., 2021)  (Duffey et al., 2021)  (Knowles, Niven, & Fawkner, 2011)  Study 3 of PhD |
| **Behavioural Regulation**  (Anything aimed at managing or changing objectively observed or measured actions) | Self-monitoring  Breaking habit  Action planning |  | (Rhodes, Perdew, & Malli, 2020)  (Rhodes et al., 2016) |

Cowley, E. S., Watson, P. M., Foweather, L., Belton, S., Thompson, A., Thijssen, D., & Wagenmakers, A. J. M. (2021). "Girls Aren't Meant to Exercise": Perceived Influences on Physical Activity among Adolescent Girls--The HERizon Project. *Children*, *8*(1), 1-16. <https://doi.org/10.3390/children8010031>

Duffey, K., Barbosa, A., Whiting, S., Mendes, R., Yordi Aguirre, I., Tcymbal, A., Abu-Omar, K., Gelius, P., & Breda, J. (2021). Barriers and Facilitators of Physical Activity Participation in Adolescent Girls: A Systematic Review of Systematic Reviews [Article]. *Frontiers in public health*, *9*, 743935. <https://doi.org/10.3389/fpubh.2021.743935>

Emm-Collison, L., Cross, R., Garcia Gonzalez, M., Watson, D., Foster, C., & Jago, R. (2022). Children’s voices in physical activity research: A qualitative review and synthesis of UK children’s perspectives. *International Journal of Environmental Research and Public Health*, *19*(7), 3993.

Ingram, G., McCormick, A., & Gibson, K. (2021). Parents’ experiences of starting and maintaining exercise: A qualitative systematic review. *Psychology of Sport and Exercise*, *57*, 102058.

Knowles, A. M., Niven, A., & Fawkner, S. (2011). A qualitative examination of factors related to the decrease in physical activity behavior in adolescent girls during the transition from primary to secondary school. *J Phys Act Health*, *8*(8), 1084-1091. <https://doi.org/10.1123/jpah.8.8.1084>

McGannon, K. R., & Schinke, R. J. (2013). “My first choice is to work out at work; then I don't feel bad about my kids”: a discursive psychological analysis of motherhood and physical activity participation. *Psychology of Sport and Exercise*, *14*(2), 179-188.

Rhodes, R. E., Berry, T., Faulkner, G., Latimer‐Cheung, A., O'Reilly, N., Tremblay, M. S., Vanderloo, L., & Spence, J. C. (2019). Application of the Multi‐Process action control framework to understand parental support of child and youth physical activity, sleep, and screen time behaviours. *Applied Psychology: Health and Well‐Being*, *11*(2), 223-239.

Rhodes, R. E., Cox, A., & Sayar, R. (2022). What predicts the physical activity intention–behavior gap? A systematic review. *Annals of Behavioral Medicine*, *56*(1), 1-20.

Rhodes, R. E., Kaushal, N., & Quinlan, A. (2016). Is physical activity a part of who I am? A review and meta-analysis of identity, schema and physical activity. *Health Psychology Review*, *10*(2), 204-225.

Rhodes, R. E., & Lim, C. (2018). Promoting parent and child physical activity together: elicitation of potential intervention targets and preferences. *Health Education & Behavior*, *45*(1), 112-123.

Rhodes, R. E., Perdew, M., & Malli, S. (2020). Correlates of Parental Support of Child and Youth Physical Activity: a Systematic Review. *Int J Behav Med*, *27*(6), 636-646. <https://doi.org/10.1007/s12529-020-09909-1>

Rhodes, R. E., Spence, J. C., Berry, T., Deshpande, S., Faulkner, G., Latimer-Cheung, A., O'Reilly, N., & Tremblay, M. S. (2016). Understanding action control of parental support behavior for child physical activity. *Health Psychology*, *35*(2), 131-140. <https://doi.org/http://dx.doi.org/10.1037/hea0000233>

Shannon, C. S. (2014). Facilitating physically active leisure for children who are overweight: Mothers' experiences. *Journal of Leisure Research*, *46*(4), 395-418.

Sohun, R., MacPhail, A., & MacDonncha, C. (2020). Physical activity parenting practices in Ireland: a qualitative analysis. *Sport, Education and Society*, 1-14. <https://doi.org/10.1080/13573322.2020.1723520>

Stirrup, J., Duncombe, R., & Sandford, R. (2015). ‘Intensive mothering’in the early years: the cultivation and consolidation of (physical) capital. *Sport, Education and Society*, *20*(1), 89-106.

Sutcliffe, J. T., Fernandez, D. K., Kelly, P. J., & Vella, S. A. (2021). The parental experience in youth sport: a systematic review and qualitative meta-study. *International Review of Sport and Exercise Psychology*, 1-28.

**Table 2.** Identification of the proposed mechanisms of action for each target behaviour

| **Target behaviour 1: Improve mothers PA support behaviours (e.g. encouragement, logistical support, co-activity, environmental and regulatory support) for their pre-teen daughters** | |
| --- | --- |
| **Mechanisms of action as per TDF domains** | **What needs to happen for change to occur?** |
| **Knowledge** | Develop mothers knowledge and understanding of PA and PA support behaviours. |
| **Skills** | Improve mothers’ skills to select and engage in PA supportive behaviours |
| **Social role and identity** | Develop mothers identity as a person who provides support for their daughters’ PA |
| **Beliefs about capabilities** | Improve perceived competence in their ability to engage in PA supportive behaviours |
| **Beliefs about consequences** | Enhance mothers’ expectations related to the positive consequences of engaging in PA supportive behaviours. |
| **Intentions** | Increase mothers’ autonomous motivation to support their daughters to be active. |
| **Goals** | Develop mothers’ use of goal setting and monitoring to facilitate engagement in selected PA support behaviours |
| **Environmental context and resources** | Provide mothers with knowledge of PA opportunities available in their area so they can support their daughter to be active. Provide mothers with materials or equipment so they can support their daughters’ PA |
| **Social influences** | Develop mothers understanding of the type of support available to them regarding supporting their daughters’ PA and their ability to engage with the support available to them |
| **Emotion** | Promote positive and reduce unpleasant emotions associated with providing PA support. |
| **Behavioural regulation** | Develop mothers ability to select and apply PA support behaviours into daily life and  implement tools to monitor PA support progress. |
| **Target behaviour 2: Increase pre-teen girls leisure time PA (outside school hours, includes active travel, sport, family activities (bike rides, walks) and outdoor play)** | |
| **Mechanisms of action as per TDF domains** | **What needs to happen for change to occur?** |
| **Knowledge** | Develop girls’ knowledge and understanding of the types and benefits of PA and how to be active. |
| **Skills** | Improve girls’ skills to be active. Provide girls with materials or equipment so they can be active |
| **Social role and identity** | Develop girls identity as a person who is physically active |
| **Beliefs about capabilities** | Improve girls’ perceived competence in their ability to engage in PA behaviours |
| **Beliefs about consequences** | Enhance girls’ expectations related to the positive consequences of engaging in PA behaviours. |
| **Intentions** | Increase girls’ autonomous motivation to support their daughters to be active. |
| **Goals** | Develop girls’ use of goal setting and monitoring to facilitate engagement in selected PA behaviours |
| **Environmental context and resources** | Provide girls with knowledge of PA opportunities available in their area so they can be active. Provide girls with materials or equipment so they can be active. |
| **Social influences** | Develop girls understanding of the type of support available to them regarding their PA, and their ability to engage with the support available to them |
| **Emotion** | Promote positive and reduce unpleasant emotions associated with being active |
| **Behavioural regulation** | Develop girls’ ability to apply PA behaviours into daily life and implement tools to monitor PA progress. |
