# Supplementary material for: Developing an Evidence- and Theory-Informed Mother-Daughter mHealth Intervention Prototype Targeting Physical Activity in Preteen Girls of Low Socioeconomic Position: Multiphase Co-Design Study
Source: JMIR Pediatr Parent. 2025 Jan 6;8:e62795. doi: 10.2196/62795 (PMC11747544; doi:10.2196/62795)
Supplement: Multimedia Appendix 4 [file pediatrics_v8i1e62795_app4.docx]

| **Co-design Workshop Mothers Group Session 2**  **Aim:** To find out mothers thoughts on app’s features and functionality, which includes look and feel, notifications, graphics, layout, usability, likes and dislikes and other reactions.  **Method:** Think aloud method guided by a script, mothers grade features of each section by using a Likert scale and add comments at the end  **Aim:** To find out mothers thoughts on app’s features and functionality, which includes look and feel, notifications, graphics, layout, usability, likes and dislikes and other reactions.  **Method:** Think aloud method guided by a script, mothers grade features of each section by using a Likert scale and add comments at the end  **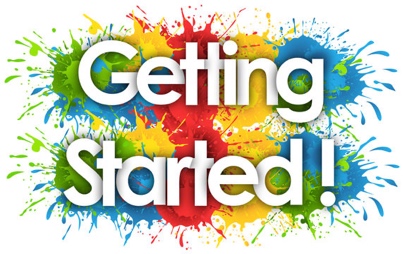Section 1**   \| **Feature** \| **Really like** \| **Like** \| **Neutral** \| **Dislike** \| **Really dislike** \| \| --- \| --- \| --- \| --- \| --- \| --- \| \| Welcome video from Carol \|  \|  \|  \|  \|  \| \| Questionnaire style \|  \|  \|  \|  \|  \| \| Number of questions \|  \|  \|  \|  \|  \| \| Language of questions \|  \|  \|  \|  \|  \| \| Smilie at the end of the section \|  \|  \|  \|  \|  \| \| **Is there anything that would put you off?** \| \| \| \| \| \| \| **What would you like changed to make it easier for you?**  **More or less text, videos, points or digital badges?** \| \| \| \| \| \|   **Section 2:**  **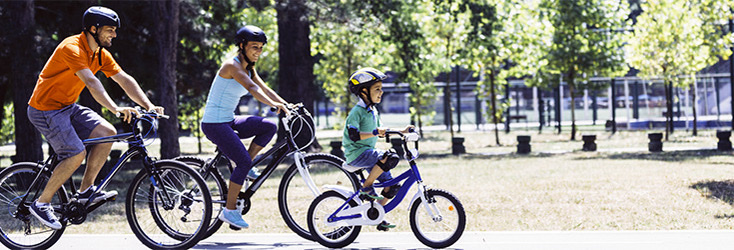**   \| **Feature** \| **Really like** \| **Like** \| **Neutral** \| **Dislike** \| **Really dislike** \| \| --- \| --- \| --- \| --- \| --- \| --- \| \| Information about physical activity \|  \|  \|  \|  \|  \| \| Pictures used to show information \|  \|  \|  \|  \|  \| \| Challenge question at the end \|  \|  \|  \|  \|  \| \|  \|  \|  \|  \|  \|  \| \| **Is there anything that would put you off?** \| \| \| \| \| \| \| **What would you like changed to make it easier for you?**  **More or less text, videos, points or digital badges?** \| \| \| \| \| \|   **Section 3**  **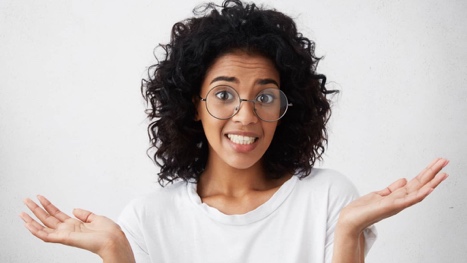**   \| **Feature** \| **Really like** \| **Like** \| **Neutral** \| **Dislike** \| **Really dislike** \| \| --- \| --- \| --- \| --- \| --- \| --- \| \| Information about supporting your daughter to be active \|  \|  \|  \|  \|  \| \| Pictures used in this section \|  \|  \|  \|  \|  \| \| Video of mother taking about support \|  \|  \|  \|  \|  \| \| Picture showing you how to find the resources \|  \|  \|  \|  \|  \| \| Challenge question at the end \|  \|  \|  \|  \|  \| \|  \|  \|  \|  \|  \|  \| \| **Is there anything that would put you off?** \| \| \| \| \| \| \| **What would you like changed to make it easier for you?**  **More or less text, videos, points or digital badges?** \| \| \| \| \| \|   **Section 4**  **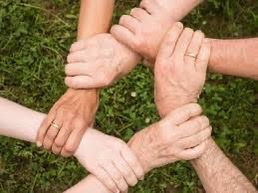**   \| **Feature** \| **Really like** \| **Like** \| **Neutral** \| **Dislike** \| **Really dislike** \| \| --- \| --- \| --- \| --- \| --- \| --- \| \| Information about how can help you support your daughter to be active \|  \|  \|  \|  \|  \| \| Pictures used in this section \|  \|  \|  \|  \|  \| \| Challenge question at the end \|  \|  \|  \|  \|  \| \|  \|  \|  \|  \|  \|  \| \| **Is there anything that would put you off?** \| \| \| \| \| \| \| **What would you like changed to make it easier for you?**  **More or less text, videos, points or digital badges?** \| \| \| \| \| \|   **Section 5**  **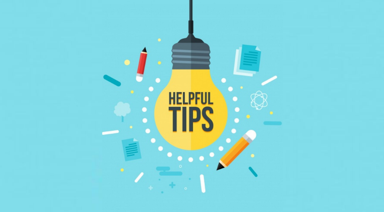**   \| **Feature** \| **Really like** \| **Like** \| **Neutral** \| **Dislike** \| **Really dislike** \| \| --- \| --- \| --- \| --- \| --- \| --- \| \| Information about tips to help you support your daughter to be active \|  \|  \|  \|  \|  \| \| Pictures used in this section \|  \|  \|  \|  \|  \| \| Video of Sonia O’ Sullivan \|  \|  \|  \|  \|  \| \|  \|  \|  \|  \|  \|  \| \| **Is there anything that would put you off?** \| \| \| \| \| \| \| **What would you like changed to make it easier for you?**  **More or less text, videos, points or digital badges?** \| \| \| \| \| \|   **Section 6**  **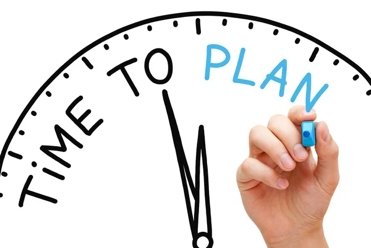**   \| **Feature** \| **Really like** \| **Like** \| **Neutral** \| **Dislike** \| **Really dislike** \| \| --- \| --- \| --- \| --- \| --- \| --- \| \| Information about next steps \|  \|  \|  \|  \|  \| \| Picking your goal \|  \|  \|  \|  \|  \| \| Track your progress \|  \|  \|  \|  \|  \| \|  \|  \|  \|  \|  \|  \| \| **Is there anything that would put you off?** \| \| \| \| \| \| \| **What would you like changed to make it easier for you?**  **More or less text, videos, points or digital badges?** \| \| \| \| \| \|   **General App Features**   \| **Feature** \| **Really like** \| **Like** \| **Neutral** \| **Dislike** \| **Really dislike** \| \| --- \| --- \| --- \| --- \| --- \| --- \| \| Home page \|  \|  \|  \|  \|  \| \| Notes \|  \|  \|  \|  \|  \| \| Resources \|  \|  \|  \|  \|  \| \| Trackers \|  \|  \|  \|  \|  \| \| Exercise logging \|  \|  \|  \|  \|  \| \| Community forum \|  \|  \|  \|  \|  \| \|  \|  \|  \|  \|  \|  \| \| **Is there anything that would put you off?** \| \| \| \| \| \| \| **What would you like changed to make it easier for you?**  **More or less text, videos, points or digital badges?** \| \| \| \| \| \|   **Overall what did you like, not like and wish could be done differently?**  **Length of time of programme? 8 Weeks?**  **Face to Face Session first to provide information about study and how to use the app?** |
| --- | --- | --- | --- | --- | --- | --- | --- | --- | --- | --- | --- | --- | --- | --- | --- | --- | --- | --- | --- | --- | --- | --- | --- | --- | --- | --- | --- | --- | --- | --- | --- | --- | --- | --- | --- | --- | --- | --- | --- | --- | --- | --- | --- | --- | --- | --- | --- | --- | --- | --- | --- | --- | --- | --- | --- | --- | --- | --- | --- | --- | --- | --- | --- | --- | --- | --- | --- | --- | --- | --- | --- | --- | --- | --- | --- | --- | --- | --- | --- | --- | --- | --- | --- | --- | --- | --- | --- | --- | --- | --- | --- | --- | --- | --- | --- | --- | --- | --- | --- | --- | --- | --- | --- | --- | --- | --- | --- | --- | --- | --- | --- | --- | --- | --- | --- | --- | --- | --- | --- | --- | --- | --- | --- | --- | --- | --- | --- | --- | --- | --- | --- | --- | --- | --- | --- | --- | --- | --- | --- | --- | --- | --- | --- | --- | --- | --- | --- | --- | --- | --- | --- | --- | --- | --- | --- | --- | --- | --- | --- | --- | --- | --- | --- | --- | --- | --- | --- | --- | --- | --- | --- | --- | --- | --- | --- | --- | --- | --- | --- | --- | --- | --- | --- | --- | --- | --- | --- | --- | --- | --- | --- | --- | --- | --- | --- | --- | --- | --- | --- | --- | --- | --- | --- | --- | --- | --- | --- | --- | --- | --- | --- | --- | --- | --- | --- | --- | --- | --- | --- | --- | --- | --- | --- | --- | --- | --- | --- | --- | --- | --- | --- | --- | --- | --- | --- | --- | --- | --- | --- | --- | --- | --- | --- | --- | --- | --- | --- | --- | --- | --- | --- | --- | --- | --- | --- | --- | --- | --- | --- | --- | --- | --- | --- | --- | --- | --- | --- | --- | --- | --- | --- | --- | --- | --- | --- | --- | --- | --- | --- | --- | --- | --- | --- | --- | --- | --- | --- | --- | --- | --- | --- | --- | --- | --- | --- | --- | --- | --- | --- | --- | --- | --- | --- | --- | --- | --- | --- | --- | --- | --- | --- | --- | --- | --- | --- | --- | --- | --- | --- | --- | --- | --- | --- | --- | --- | --- | --- | --- | --- | --- |

| **Co-design Workshop Girls Group Session 2**  **Aim:** To find out girls thoughts on app’s features and functionality, which includes look and feel, notifications, graphics, layout, usability, likes and dislikes and other reactions.  **Method:** Think aloud method guided by a script, girls grade features of each section by using a Likert scale and add comments at the end  **Section 1**  **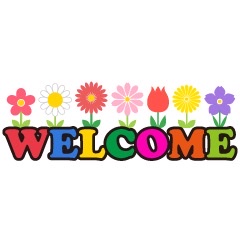**   \| **Feature** \| **Really like** \| **Like** \| **Neutral** \| **Dislike** \| **Really dislike** \| \| --- \| --- \| --- \| --- \| --- \| --- \| \| Welcome video from Carol \|  \|  \|  \|  \|  \| \| Questionnaire style \|  \|  \|  \|  \|  \| \| Number of questions \|  \|  \|  \|  \|  \| \| Language of questions \|  \|  \|  \|  \|  \| \| Smilie at the end of the section \|  \|  \|  \|  \|  \| \|  \|  \|  \|  \|  \|  \| \| **Is there anything that would put you off?** \| \| \| \| \| \| \| **What would you like changed to make it easier for you?**  **More or less text, videos, points or digital badges?** \| \| \| \| \| \|   **Section 2:**  **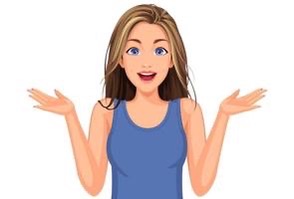**   \| **Feature** \| **Really like** \| **Like** \| **Neutral** \| **Dislike** \| **Really dislike** \| \| --- \| --- \| --- \| --- \| --- \| --- \| \| Information about being active \|  \|  \|  \|  \|  \| \| Pictures used to show information \|  \|  \|  \|  \|  \| \| Challenge question at the end \|  \|  \|  \|  \|  \| \|  \|  \|  \|  \|  \|  \| \| **Is there anything off put you off?** \| \| \| \| \| \| \| **What would you like changed to make it easier for you?**  **More or less text, videos, points or digital badges?** \| \| \| \| \| \|   **Section 3**  **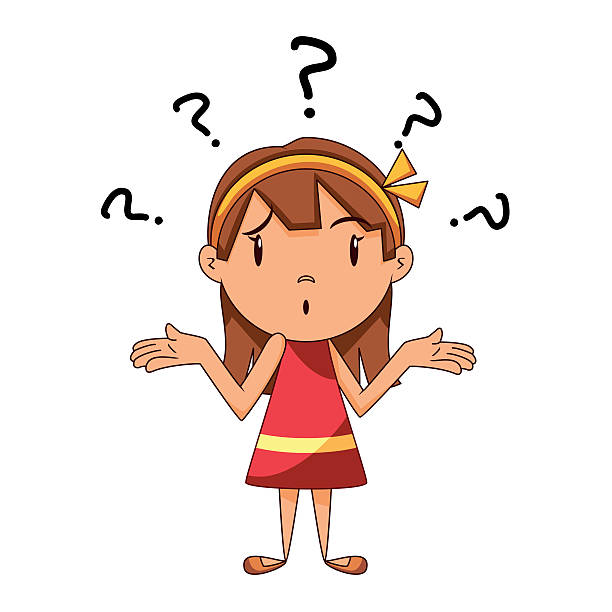**   \| **Feature** \| **Really like** \| **Like** \| **Neutral** \| **Dislike** \| **Really dislike** \| \| --- \| --- \| --- \| --- \| --- \| --- \| \| Information about why we should be active \|  \|  \|  \|  \|  \| \| Pictures used in this section \|  \|  \|  \|  \|  \| \| Video about what is good about being active \|  \|  \|  \|  \|  \| \| Video of Alice \|  \|  \|  \|  \|  \| \| Challenge question at the end \|  \|  \|  \|  \|  \| \|  \|  \|  \|  \|  \|  \| \| **Is there anything off put you off?** \| \| \| \| \| \| \| **What would you like changed to make it easier for you?**  **More or less text, videos, points or digital badges?** \| \| \| \| \| \|   **Section 4**  **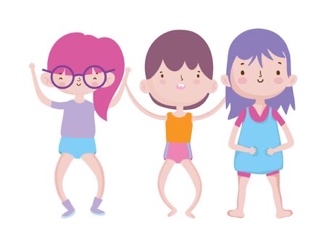**   \| **Feature** \| **Really like** \| **Like** \| **Neutral** \| **Dislike** \| **Really dislike** \| \| --- \| --- \| --- \| --- \| --- \| --- \| \| Information about how to be active \|  \|  \|  \|  \|  \| \| Pictures used in this section \|  \|  \|  \|  \|  \| \| Video of what you can do to be active \|  \|  \|  \|  \|  \| \| Video about Katie Mc Cabe \|  \|  \|  \|  \|  \| \| Survey question at the end \|  \|  \|  \|  \|  \| \|  \|  \|  \|  \|  \|  \| \| **Is there anything off put you off?** \| \| \| \| \| \| \| **What would you like changed to make it easier for you?**  **More or less text, videos, points or digital badges?** \| \| \| \| \| \|   **Section 5**  **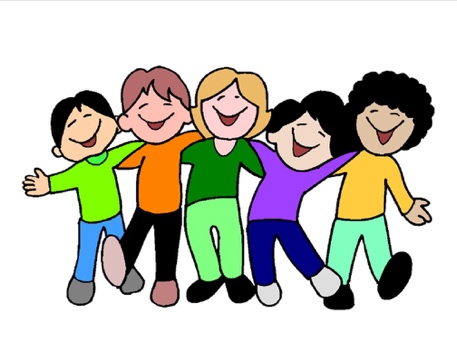**   \| **Feature** \| **Really like** \| **Like** \| **Neutral** \| **Dislike** \| **Really dislike** \| \| --- \| --- \| --- \| --- \| --- \| --- \| \| Information about who you can be active with \|  \|  \|  \|  \|  \| \| Pictures used in this section \|  \|  \|  \|  \|  \| \| Challenge question at the end \|  \|  \|  \|  \|  \| \|  \|  \|  \|  \|  \|  \| \| **Is there anything that would put you off?** \| \| \| \| \| \| \| **What would you like changed to make it easier for you?**  **More or less text, videos, points or digital badges?** \| \| \| \| \| \|   **Section 6**  **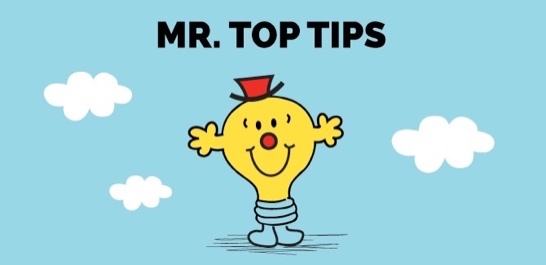**   \| **Feature** \| **Really like** \| **Like** \| **Neutral** \| **Dislike** \| **Really dislike** \| \| --- \| --- \| --- \| --- \| --- \| --- \| \| Information about tips to help you be active \|  \|  \|  \|  \|  \| \| Pictures used in this section \|  \|  \|  \|  \|  \| \| Video about girls who kept playing \|  \|  \|  \|  \|  \| \| Video about Katie Mc Cabe’s role models \|  \|  \|  \|  \|  \| \| Survey questions at the end \|  \|  \|  \|  \|  \| \|  \|  \|  \|  \|  \|  \| \| **Is there anything that would put you off?** \| \| \| \| \| \| \| **What would you like changed to make it easier for you?**  **More or less text, videos, points or digital badges?** \| \| \| \| \| \|   **Section 7**  **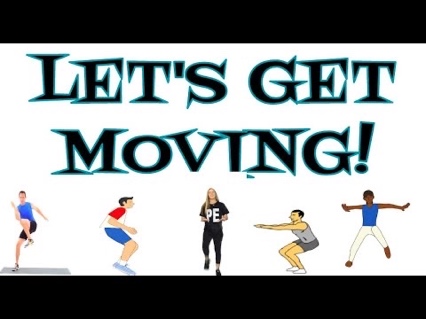**   \| **Feature** \| **Really like** \| **Like** \| **Neutral** \| **Dislike** \| **Really dislike** \| \| --- \| --- \| --- \| --- \| --- \| --- \| \| Information about Let’s Get Moving \|  \|  \|  \|  \|  \| \| Pictures used in this section \|  \|  \|  \|  \|  \| \| Goal setting part \|  \|  \|  \|  \|  \| \| **Track your progress** \|  \|  \|  \|  \|  \| \|  \|  \|  \|  \|  \|  \| \| **Is there anything off put you off?** \| \| \| \| \| \| \| **What would you like changed to make it easier for you?**  **More or less text, videos, points or digital badges?** \| \| \| \| \| \|   **General App Features**   \| **Feature** \| **Really like** \| **Like** \| **Neutral** \| **Dislike** \| **Really dislike** \| \| --- \| --- \| --- \| --- \| --- \| --- \| \| Home page \|  \|  \|  \|  \|  \| \| Notes \|  \|  \|  \|  \|  \| \| Resources \|  \|  \|  \|  \|  \| \| Trackers \|  \|  \|  \|  \|  \| \| Scored surveys \|  \|  \|  \|  \|  \| \| My points \|  \|  \|  \|  \|  \| \| Exercise logging \|  \|  \|  \|  \|  \| \| Community forum \|  \|  \|  \|  \|  \| \|  \|  \|  \|  \|  \|  \| \| **Is there anything that would put you off?** \| \| \| \| \| \| \| **What would you like changed to make it easier for you?**  **More or less text, videos, points or digital badges?** \| \| \| \| \| \|   **Overall what did you like, not like and wish could be done differently?** |
| --- | --- | --- | --- | --- | --- | --- | --- | --- | --- | --- | --- | --- | --- | --- | --- | --- | --- | --- | --- | --- | --- | --- | --- | --- | --- | --- | --- | --- | --- | --- | --- | --- | --- | --- | --- | --- | --- | --- | --- | --- | --- | --- | --- | --- | --- | --- | --- | --- | --- | --- | --- | --- | --- | --- | --- | --- | --- | --- | --- | --- | --- | --- | --- | --- | --- | --- | --- | --- | --- | --- | --- | --- | --- | --- | --- | --- | --- | --- | --- | --- | --- | --- | --- | --- | --- | --- | --- | --- | --- | --- | --- | --- | --- | --- | --- | --- | --- | --- | --- | --- | --- | --- | --- | --- | --- | --- | --- | --- | --- | --- | --- | --- | --- | --- | --- | --- | --- | --- | --- | --- | --- | --- | --- | --- | --- | --- | --- | --- | --- | --- | --- | --- | --- | --- | --- | --- | --- | --- | --- | --- | --- | --- | --- | --- | --- | --- | --- | --- | --- | --- | --- | --- | --- | --- | --- | --- | --- | --- | --- | --- | --- | --- | --- | --- | --- | --- | --- | --- | --- | --- | --- | --- | --- | --- | --- | --- | --- | --- | --- | --- | --- | --- | --- | --- | --- | --- | --- | --- | --- | --- | --- | --- | --- | --- | --- | --- | --- | --- | --- | --- | --- | --- | --- | --- | --- | --- | --- | --- | --- | --- | --- | --- | --- | --- | --- | --- | --- | --- | --- | --- | --- | --- | --- | --- | --- | --- | --- | --- | --- | --- | --- | --- | --- | --- | --- | --- | --- | --- | --- | --- | --- | --- | --- | --- | --- | --- | --- | --- | --- | --- | --- | --- | --- | --- | --- | --- | --- | --- | --- | --- | --- | --- | --- | --- | --- | --- | --- | --- | --- | --- | --- | --- | --- | --- | --- | --- | --- | --- | --- | --- | --- | --- | --- | --- | --- | --- | --- | --- | --- | --- | --- | --- | --- | --- | --- | --- | --- | --- | --- | --- | --- | --- | --- | --- | --- | --- | --- | --- | --- | --- | --- | --- | --- | --- | --- | --- | --- | --- | --- | --- | --- | --- | --- | --- | --- | --- | --- | --- | --- | --- | --- | --- | --- | --- | --- | --- | --- | --- | --- | --- | --- | --- | --- | --- | --- | --- | --- | --- | --- | --- | --- | --- | --- | --- | --- | --- | --- | --- | --- | --- | --- | --- | --- | --- | --- | --- | --- | --- | --- | --- | --- | --- | --- | --- | --- | --- | --- | --- | --- | --- | --- | --- | --- | --- | --- | --- | --- | --- | --- | --- | --- | --- | --- | --- | --- | --- | --- | --- | --- | --- | --- | --- | --- | --- | --- | --- | --- | --- | --- | --- | --- | --- | --- | --- | --- | --- | --- | --- | --- | --- |
