# Supplementary material for: Developing an Evidence- and Theory-Informed Mother-Daughter mHealth Intervention Prototype Targeting Physical Activity in Preteen Girls of Low Socioeconomic Position: Multiphase Co-Design Study
Source: JMIR Pediatr Parent. 2025 Jan 6;8:e62795. doi: 10.2196/62795 (PMC11747544; doi:10.2196/62795)
Supplement: Multimedia Appendix 5 [file pediatrics_v8i1e62795_app5.docx]

Behaviour change potential of PAVA app – App Behaviour Change Scale (McKay et al., 2019)

| **Scale item number and question** | **Definition** | **Final Rating^a^** |
| --- | --- | --- |
| **1 Knowledge and information** |  |  |
| **1.1** Does the app have the ability to customize and personalize some features? | Elements of the app can be personalized through specific tools or functions that are specific to the individual using the app. | 1 |
| **1.2**  Was the app created with expertise and/or Does the app provide information that is consistent with national guidelines? | This would be found in the about section or generally in the app. | 1 |
| **1.3** Does the app ask for baseline in- formation? | This includes BMI, weight, smoking rate, exercise, or drinking behaviours | 1 |
| **1.4** Does the app provide instruction on how to perform the behaviour? | The app is clear in telling the person how to perform a behaviour or preparatory behaviours, either verbally, through video, or in written form. NB: the behaviour that is seeking to be changed, not information on how to use the app | 1 |
| **1.5** Does the app provide information about the consequences of continuing and/or discontinuing behaviour? | The app gives the user information about the consequences of behaviour in general, this includes information about the relationship between the behaviour and its possible or likely consequences in the general case. This information can be general or personalized. | 1 |
| 1. **Goals and planning** |  |  |
| **2.1** Does the app ask for willingness for behaviour change? | Is there a feature during setup where you describe how ready you are for behaviour change? | 0 |
| **2.2** Does the app allow for the setting of goals? | The person is encouraged to make a behavioural resolution.  The person is encouraged to set a general goal that can be achieved by behavioural means. This includes subgoals or preparatory behaviours and/or specific contexts in which the behaviour will be performed. The behaviour in this technique will be directly related to or be a necessary condition for the target behaviour | 1 |
| **2.3** Does the app have the ability to review goals, update, and change when necessary? | Involves a review or analysis of the extent to which previously set behavioural goals (regardless of short or long) were achieved. | 1 |
| 1. **Feedback and monitoring** |  |  |
| **3.1** Does the app give the user the ability to quickly and easily understand the difference between current action and future goals? | Allows user to see how they are tracking against a goal and to see the difference between what they want to do and what they are currently doing. This will give some feedback on where they are at and what they need to change to get to where they want to be. | 1 |
| **3.2** Does the app have the ability to allow the user to easily self- monitor behaviour? | The app allows for a regular monitoring of the activity. | 1 |
| **3.3** Does the app have the ability to share behaviours with others (including social media or forums) and/or allow for social comparison? | The app allows the person to share his or her behaviours on social media or in forums. This could also include a *buddy* system or a leader board | 1 |
| **3.4** Does the app have the ability to give the user feedback—either from a person or automatically? | The app is able to provide the person with feedback, comments, or data about their own recorded behaviour. This might be automatic or could be personal. | 1 |
| **3.5** Does the app have the ability to export data from app? | The app allows for the export of information and progress to an external user. | 0 |
| **3.6** Does the app provide a material or social reward or incentive | App provides rewards for attempts at achieving a behavioural goal. This might include efforts made toward achieving the behaviour or progress made in preparatory steps toward the behaviour or in achieving a goal. | 1 |
| **3.7** Does the app provide general encouragement? | The app provides general encouragement and positive reinforcement on actions leading to the goal. | 1 |
| 1. **Actions** |  |  |
| **4.1** Does the app have reminders and/or prompts or cues for activity? | The app prompts the user to engage in the activity. The app has the ability to give notifications or reminders to cue the behaviour. | 1 |
| **4.2** Does the app encourage positive habit formation? | The app prompts explicit rehearsal and repetition of the behaviour–not just tracking or logging. | 1 |
| **4.3** Does the app allow or encourage for practice or rehearsal, in addition to daily activities? | App does not have a lock on activities or a number that you cannot exceed daily. | 1 |
| **4.4** Does the app provide opportunity to plan for barriers? | The app encourages the person to think about potential barriers and identify ways of overcoming them. | 1 |
| **4.5** Does the app assist with or suggest restructuring the physical or social environment? | The app prompts the person to alter the environment in ways so that it is more supportive of the target behaviour. | 1 |
| **4.6** Does the app assist with distraction or avoidance? | The app gives suggestions and advice on how the person can avoid situations or distract themselves when trying to reach their goal. | 0 |
| **Total score** |  | **18** |

McKay, F. H., Slykerman, S., & Dunn, M. (2019). The app behavior change scale: creation of a scale to assess the potential of apps to promote behavior change. *JMIR mHealth and uHealth*, *7*(1), e11130.
