# Supplementary material for: Developing an Evidence- and Theory-Informed Mother-Daughter mHealth Intervention Prototype Targeting Physical Activity in Preteen Girls of Low Socioeconomic Position: Multiphase Co-Design Study
Source: JMIR Pediatr Parent. 2025 Jan 6;8:e62795. doi: 10.2196/62795 (PMC11747544; doi:10.2196/62795)

Aim of workshop

To meet with mothers of girls aged 10-12 years and get their input to help us design a programme to help mothers support their daughters to be physically active.

What will we be doing?

- Carol and James will provide a brief introduction to the study.
- Carol and James will provide information about physical activity, supporting girls to be active and what needs to be done to help mothers support their daughters.
- In small groups everyone will work together to describe how might we help mothers support their daughters.
- In small groups everyone will work together to describe and discuss what tools can be used to help mothers.
- In small groups everyone will work together to describe and discuss how and when when can deliver strategies to mothers.
- Finish with group discussion about advantages and disadvantages of the strategies and the next steps in the study.

What does leisure time physical activity mean?

It means taking part in physical activity outside of work or school hours. It includes sports participation, exercise or training, active travel, and recreational activities such as going for a walk, dancing or gardening.

What are the benefits of being active?

- ✓ Health benefits for our hearts, bodies and minds.
- ✓ Helps us think and learn better
- ✓ Time with our family and friends

How much physical activity is needed for health?

Adults/Older Adults

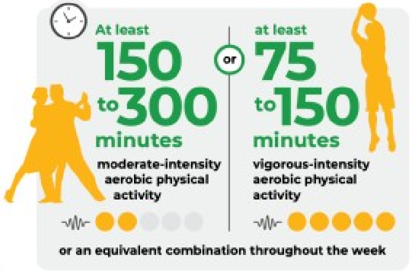

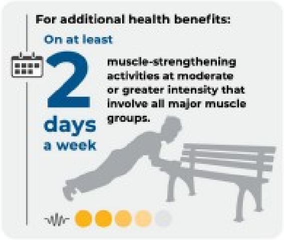

Children and Adolescents

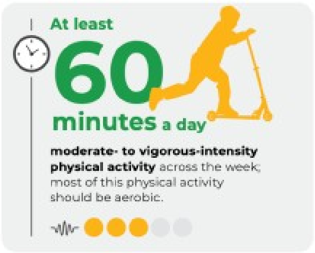

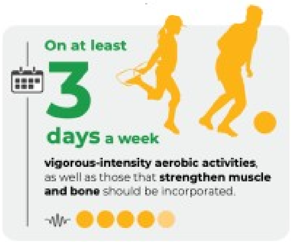

How active are our children and teenagers?

81% of Irish teenage girls (11-17 years) and 64% of Irish teenage boys (11-17 years) do not meet the recommended amount of physical activity for health.

Girls are less active than boys, and even more so at the weekends or during school holidays. Many girls drop out of sport or activities as they progress to secondary school.

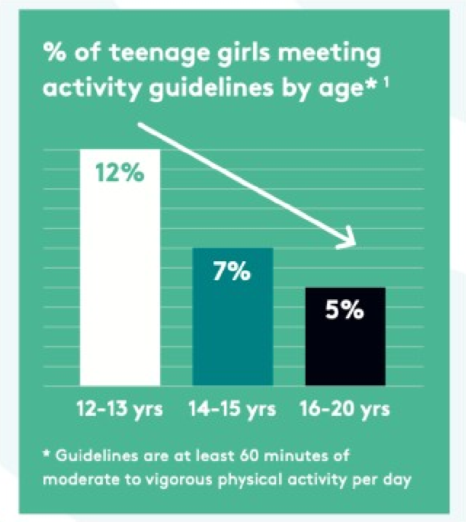

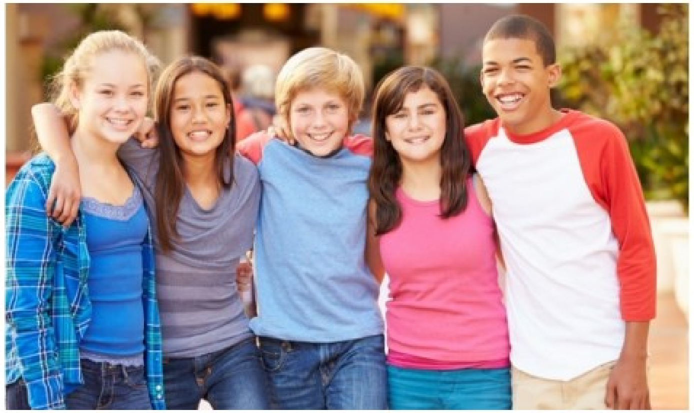

How can a mother support their daughter to be active?

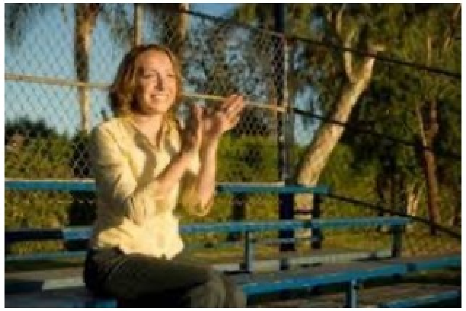

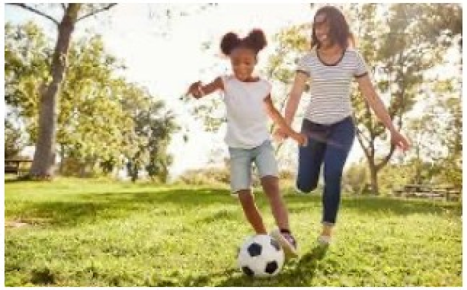

There are many ways that a mother can support her daughter to be active:

- ✓ Encouragement
- ✓ Praise
- ✓ Positive feedback
- ✓ Arranging transport
- ✓ Being active with their daughter
- ✓ Enrol daughter in activities
- ✓ Provide equipment and materials necessary.
- ✓ Establishing rules and regulations around being active.

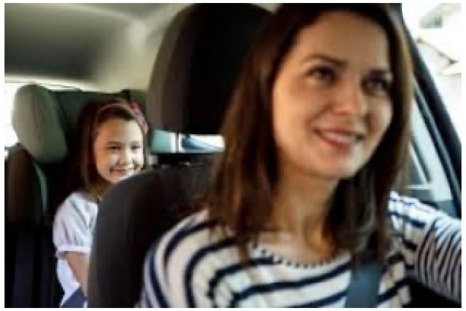

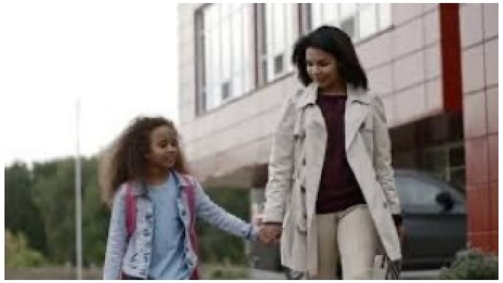

What challenges do mothers face when supporting their daughters to be active?

I'm not very sporty

She has no friends there

I have to work

She has no interest in it

My husband/ partner does all the sports activities

It is a battle to get her there

I was not very good at sports when I was younger

Anti-social behaviour in the playground

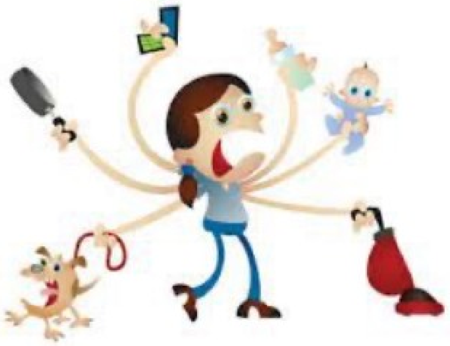

Too many family fights when we are active together

The roads are too dangerous

She doesn't want to go

It's very expensive

It's hard to find the time

We have very little choices around here

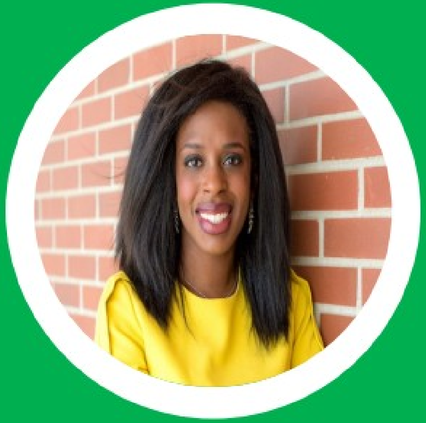

Debbie

Age:	37 years old
Marital status:	Living with partner
Location:	City
Children:	Girl aged 11 and boy aged 14
Employment:	Stay-at-home parent

Debbie did not play any sport in her youth. Debbie feels that is important that her children play sport. Her daughter enjoys athletics and basketball in school, and Debbie hopes she will try new sports when she goes to secondary school.

Debbie's story

- Debbie did not play any sport and did not like PE in school
- She enjoys going for walks when the weather is nice. Her daughter comes too sometimes.
- Would like her daughter to try new sports in secondary school.
- Would like to try some new activities for herself.

Challenges Debbie faces:

- Home life is hectic for Debbie as she has the children and home responsibilities.
- Outside of school setting is unsure about involving her daughter in sports clubs, especially as she doesn't drive.
- Lacks confidence in her ability to be active or take up new hobbies, and likes music.
- Is concerned about anti-social behaviour in the playgrounds and green in their estate.

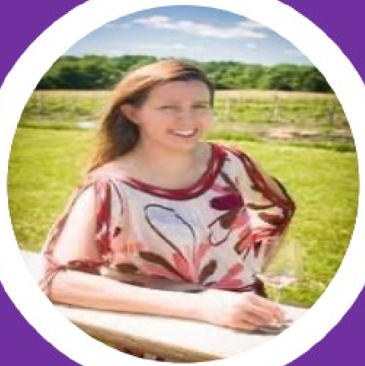

Sandra

Age:	43 years old
Marital status:	Married
Location:	Small town
Children:	2 boys (ages 5 and7), 1 girl (age 11)
Employment:	Part time, 3 days a week

The local GAA club is an important part of Sandra's life, it is where she met her husband and friends. Sandra would like her daughter to have some of the positive experiences she had through playing sport.

Sandra's story

- Sandra played camogie for her county in her youth
- Helps coach her daughter's U12's team at her local GAA club.
- Enjoys going for a run with one of her friends, when she can.
- Enjoys going for walks/ cycles with her family when they have time.

Challenges Sandra faces:

- Very busy household, husband works full time, finds it difficult to get time to go for a run or arrange family walks/cycles.
- When GAA season is over, difficult to encourage daughter to be active regularly
- Is nervous of the main road that is near their home
- Hard to find something to suit all the family.

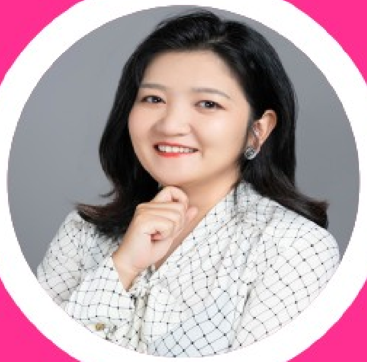

Ruth

Age: 42 years old
Marital status: Single
Location: City
Children: Girl aged 12, boy age 6
Employment: Works full time

Ruth played soccer for her school team but was not part of a club. She feels it is important to lead by example and tries to go to the gym when she can. Her children are part of the local GAA and athletics clubs.

Ruth's story

- Ruth enjoyed playing soccer for her school team and liked PE in school and regrets not joining a club.
- Ruth likes to go to the gym when she can find time.
- Would like her daughter to continue to be part of a sports club as she gets older.
- Would like to try some new activities for herself.

Challenges Ruth faces:

- Juggling work commitments and family life is difficult.
- Some financial pressure due to circumstances and current economic climate.
- Finds it challenging to organise family walks/ hikes/ bike rides.
- Is concerned about anti-social behaviour in the playgrounds and green in their estate.

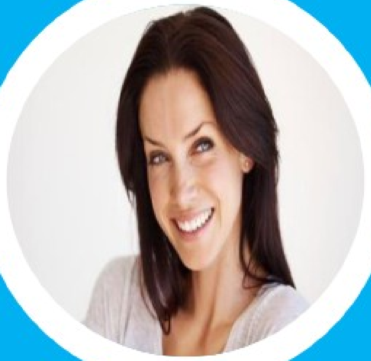

Karen

Age:	39 years old
Marital status:	Separated
Location:	Medium sized town
Children:	2 girls (ages 10 and 12)
Employment:	Works full time

Karen was Irish dancer up to the age of 12, but did not take up any sports after that. Karen did not enjoy PE in school. She would like her daughters to play team sports but is unsure of how to get them involved.

Karen's story

- Karen has won medals for Irish dancing in her youth
- She enjoys watching her daughters dance at competitions
- Would like to have a hobby for herself e.g. dancing
- Enjoys spending time going for walks with her daughters and the dog in the local park.

Challenges Karen faces:

- Juggling work commitments and family life is difficult.
- Some financial pressure due to circumstances and current economic climate.
- Lacks confidence in her ability to be active or take up new hobbies, and doesn't like running.
- Would like to be more involved in her daughters sports activities but is unsure of how to get involved or what contribution she could make.

How might we help mothers support their daughters?

	Examples	Rank
How can we improve mothers' knowledge and understanding about supporting their daughter to be active?		
How can we persuade mothers to support their daughter to be active?		
What would encourage or incentivise mothers to support and continue to support their daughter to be active?		
Who could mothers of pre-teen girls look up to regarding supporting their daughters?		
Who could help mothers provide support for her daughters activities?		
How could mothers learn the skills necessary to support their daughters?		

How and when can we help mothers support their daughters

How	Rank
Online training programme (completed in your own time)	
Online training programme (completed as part of a group, e.g., zoom sessions)	
Face-to-face group sessions	
Online group support (WhatsApp, Facebook)	
Resources (website, Facebook group, Instagram page, handbook, worksheets, app)	
Combination of online training programme, face-to-face group sessions and resources	
Combination of online group training programme and handbook	
Other combination of methods of delivery	

How long	Rank
8-12 weeks (once a week)	
8-12 weeks (with a six month follow up)	
6 months (once a week for 8-12 weeks, then monthly follow up)	
12 months (once a week for 8-12 weeks, monthly follow up for 6 months, 2 month follow up then)	

When	Rank
At the start of the year – January	
After Easter holidays – April	
In summer – June or July	
At the start of school year – September	
Over winter – November or December	

Next steps

April ‘23: Carol and James look back over the feedback from the workshop with mothers.

May ‘23: Carol and James consult with other experts and make revisions to the study.

June ‘23: Carol and James meet again with mothers to show them revisions and get some more feedback.

July ‘23: Carol and James look back over the feedback from the 2nd workshop with mothers.

Sept ‘23: Carol and James meet with other experts and finalise the strategies.

Oct –Jan ‘23: Carol and James write a document about the strategies and look to progress it to the next stage of development.

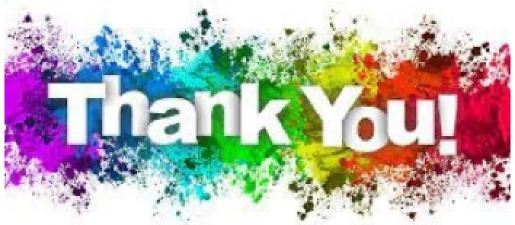

Aim of workshop

To meet with girls aged 10-12 years and get their input to help us design a programme to help girls aged 10-12 years be physically active.

What will we be doing?

- Following ice-breaker and introductions, Carol and James and provide a brief introduction to the study.
- Carol and James will provide information about physical activity and
- Carol and James will host group discussion about why girls enjoy or do not enjoy being active.
- Carol and James will introduce personas
- In small groups everyone will work together to describe how might we help girls be more active.
- Finish with group discussion about advantages and disadvantages of the strategies and the next steps in the study.

What does leisure time physical activity mean?

It means taking part in physical activity outside of school hours. It includes sports participation, outdoor play, active travel, and recreational activities such as going for a walk, cycle or dancing.

What are the benefits of being active?

- ✓ Health benefits for our hearts, bodies and minds.
- ✓ Helps us think and learn better
- ✓ Time with our family and friends

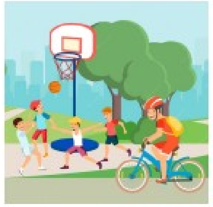

How much physical activity is needed for health?

Children and Adolescents

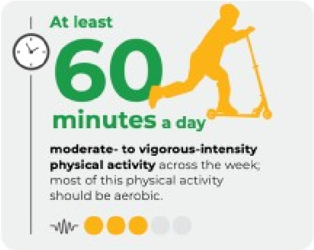

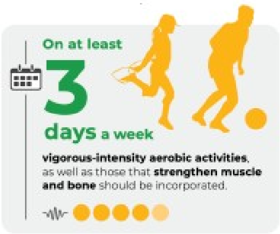

How active are children and teenagers?

81% of Irish teenage girls (11-17 years) and 64% of Irish teenage boys (11-17 years) do not meet the recommended amount of physical activity for health.

Girls are less active than boys, and even more at the weekends or out or during school holidays. Many girls drop out of sport or activities as they progress to secondary school.

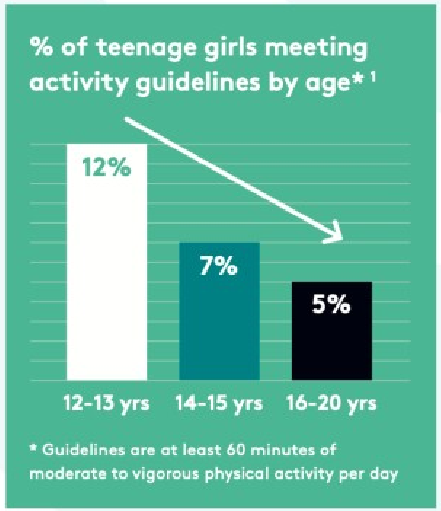

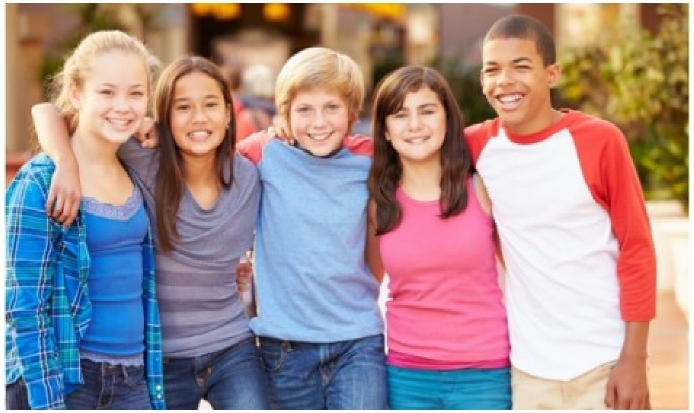

What do some girls like about being active?

It's really fun

My friends are there

I enjoy being part of a team

I enjoy spending time with my mother/ father

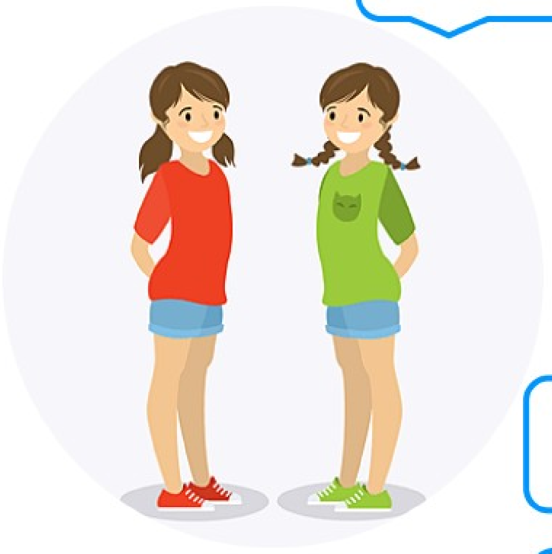

The coaches are really helpful

I like making new friends

I like playing outside with my friends

I like being competitive

It's what my family do

I like playing in the rain

I like to see how I can improve my skills

I like winning

I like when my mother or father come to watch

Why do some girls not want to be active?

I'm not very sporty

I don't like losing

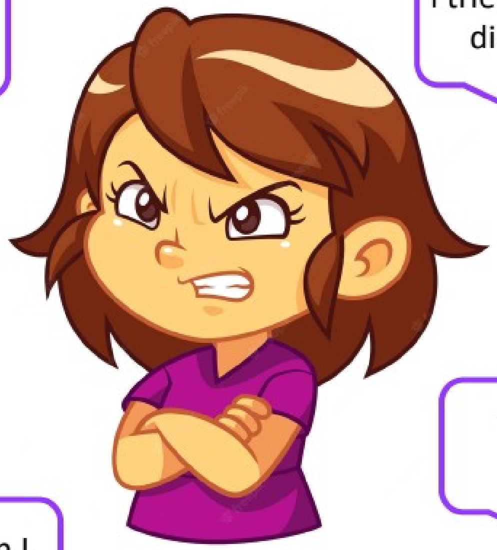

I tried before and didn't like it

I don't like being out in the rain

I have no friends there

The coaches shout a lot

I'm afraid others will laugh at me

I'm too tired

I'm no good at sports

The boys are too rough

The roads are too dangerous

I didn't start when I was very young

I have too much homework

It gets too competitive at times

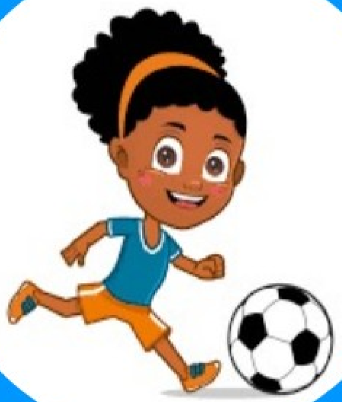

Daisy

About Daisy

Age:	11 years old
Family:	Parents, 1 brother aged 14
Lives:	In a city

What Daisy likes

Soccer and GAA, plays for her school and local club
Playing outside with her friends
Music
Xbox

Daisy's Frustrations

Boys who will not pass the ball
Coaches who shout at her
Going outside in the rain

Daisy's Goals

Wants to play Soccer for Ireland
Wants to be a Doctor

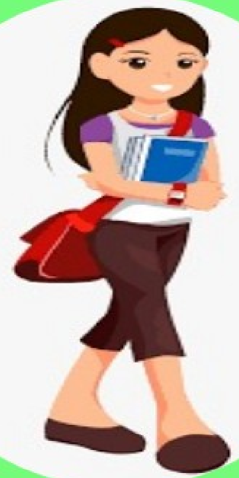

Grace

About Grace

Age:	12 years old
Family:	Parents, 1 sister aged 10, dog
Lives:	In the countryside

What Grace likes

Playing outside with her friends
Reading
Arts and crafts
Shopping

Grace's Frustrations

Tried playing basketball but did not have any friends there
She does not like PE in school
Her mother is always trying to get her to walk places

Grace's Goals

Wants to be a famous fashion designer
Wants to have lots of clothes

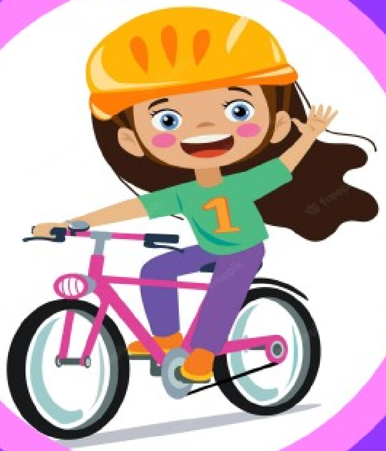

Ava

About Ava

Age: 11 years old

Family: Parents, twin sister, 1 step-brother aged 2, step-dad

Lives: In a town

What Ava likes

Cycling her bike with her friends

Her PlayStation

Singing and dancing, is in a local stage school

Going on holidays

Ava's Frustrations

Cleaning her bedroom

Older children in the park can be intimidating

Would like more time on her PlayStation

Ava's Goals

Wants to be a famous singer

Wants to travel to lots of countries

How might we help girls be more active?

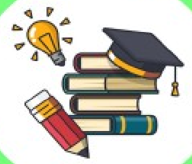

How can we help girls know more about being active?

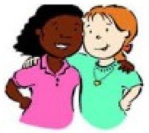

How can we persuade girls to be active?

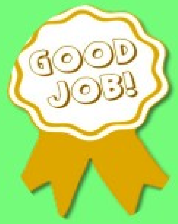

What would encourage or motivate girls to be more active?

How might we help girls be more active?

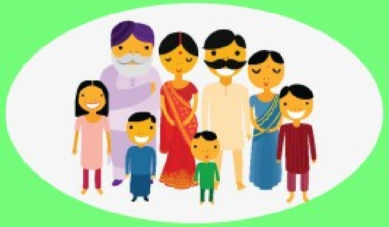

Who could support girls to take part in their activities?

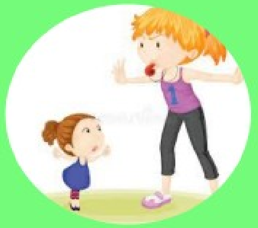

How could girls learn the skills they need to be more active?

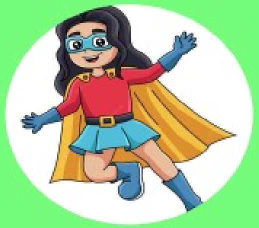

To encourage girls to be more active, who could they look up to or admire?

How can we help girls learn more about being active?

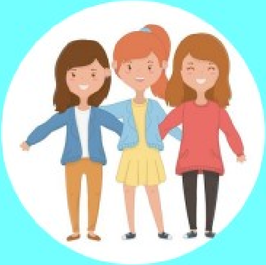

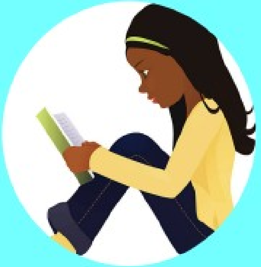

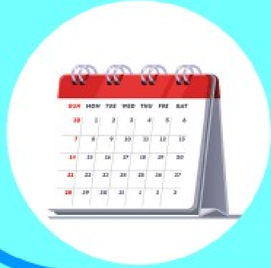

How	Yes	No	Maybe
Take part in some activities with girls their own age			
Take part in some activities with a parent, for example mother			
Get a workbook with information about how and where to active.			
An app to learn about how and where to be active.			

When would be the best time for a programme to start?

When	Yes	No	Maybe
At the start of the year – January			
After Easter holidays – April			
In summer – June or July			
At the start of school year – September			
Over winter – November or December			

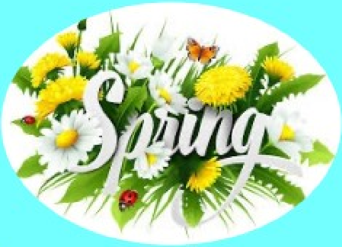

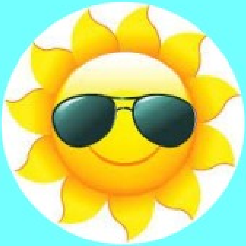

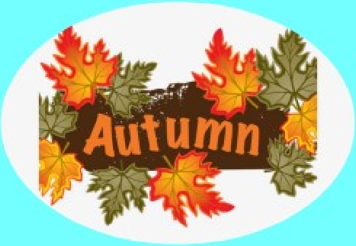

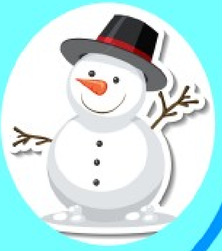

Next steps

April ‘23: Carol and James look back over the feedback from the workshops.

May ‘23: Carol and James consult with other experts and make revisions to the study.

June ‘23: Carol and James meet again with mothers to show them revisions and get some more feedback.

July ‘23: Carol and James look back over the feedback from the 2nd workshops

Sept ‘23: Carol and James meet with other experts and finalise the strategies.

Oct –Jan ‘23: Carol and James write a document about the strategies and look to progress it to the next stage of development.

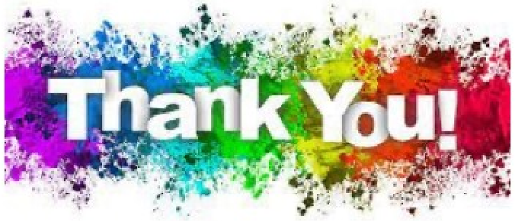

Supplement: Supplementary file 5 [file pediatrics_v8i1e62795_app3.docx]
